# Investigating Membrane‐Mediated Antimicrobial Peptide Interactions with Synchrotron Radiation Far‐Infrared Spectroscopy

**DOI:** 10.1002/cphc.202100815

**Published:** 2022-01-14

**Authors:** Andrea Hornemann, Diane M. Eichert, Arne Hoehl, Brigitte Tiersch, Gerhard Ulm, Maxim G. Ryadnov, Burkhard Beckhoff

**Affiliations:** ^1^ Department 7.1 Radiometry with Synchrotron Radiation and Department 7.2 X-Ray Metrology with Synchrotron Radiation Physikalisch-Technische Bundesanstalt (PTB) Abbestr. 2–12 10587 Berlin Germany; ^2^ ELETTRA – Sincrotrone Trieste S.S.14 Km 163.5 in Area Science Park 34149 Basovizza, Trieste Italy; ^3^ Universität Potsdam Karl-Liebknecht-Str. 24–25 14476 Potsdam Germany; ^4^ National Physical Laboratory Hampton Rd Teddington Middlesex TW11 0LW UK

**Keywords:** antimicrobial peptides, electrostatic interactions, IR spectroscopy, phospholipid membranes, protein folding

## Abstract

Synchrotron radiation‐based Fourier transform infrared spectroscopy enables access to vibrational information from mid over far infrared to even terahertz domains. This information may prove critical for the elucidation of fundamental bio‐molecular phenomena including folding‐mediated innate host defence mechanisms. Antimicrobial peptides (AMPs) represent one of such phenomena. These are major effector molecules of the innate immune system, which favour attack on microbial membranes. AMPs recognise and bind to the membranes whereupon they assemble into pores or channels destabilising the membranes leading to cell death. However, specific molecular interactions responsible for antimicrobial activities have yet to be fully understood. Herein we probe such interactions by assessing molecular specific variations in the near‐THz 400–40 cm^−1^ range for defined helical AMP templates in reconstituted phospholipid membranes. In particular, we show that a temperature‐dependent spectroscopic analysis, supported by 2D correlative tools, provides direct evidence for the membrane‐induced and folding‐mediated activity of AMPs. The far‐FTIR study offers a direct and information‐rich probe of membrane‐related antimicrobial interactions.

## Graphical abstract

**Synchrotron radiation far‐infrared spectroscopy** enables studying the mechanistic behaviour of antimicrobial peptides in reconstituted membranes, and monitoring membrane‐mediated antimicrobial interactions. Membrane polarity, antimicrobial peptide membrane‐mediated conformation, and related electrostatic interactions are key aspects leading to membrane destabilisation.

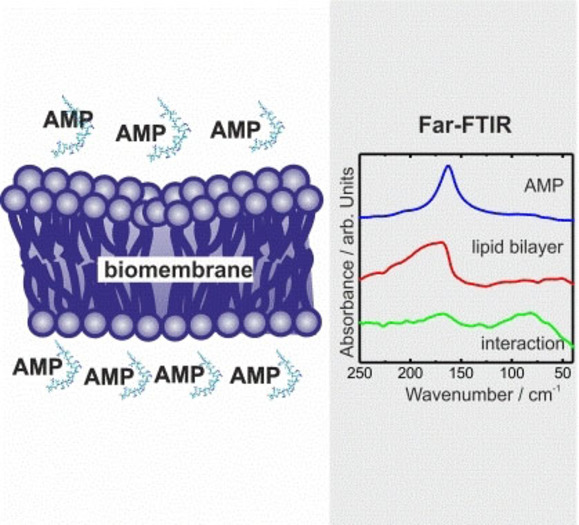

## Introduction

Peptides are naturally occurring biological macromolecules, and are chains of covalently linked amino acids. The chain's spatial arrangement, also called conformation, strongly correlates with the functional properties of peptides. Antimicrobial peptides (AMPs) are endogenous polypeptide antibiotics found in all multicellular organisms as innate host defence against pathogens. These peptides are believed to preferentially bind to microbial membranes, in which they assemble into membrane‐disrupting pores and lesions (‘carpet model’).[^1^, ^2^] For bacteria to develop resistance against such membrane‐targeting agents remains a challenge, which prompts the development of AMPs as promising antimicrobial agents in the post‐antibiotic era.^[3]^ But, AMPs are not free of certain drawbacks, including potential toxicity, susceptibility to proteases, spontaneous or induced structural plasticity[^4^, ^5^] and high cost of production, limiting their commercialisation and systemic use in clinic. While extensive attempts have been made to overcome such obstacles, main lines of research focus on studying the interrelationships between the biological activity of AMPs, their native structure, and conformational preferences in presence of a membrane, as well as their effective membrane binding,^[6]^ in order to provide clinically relevant formulations.^[7]^ Density functional theory simulations, and combination of deep learning algorithms and molecular dynamics constitute promising tools for the development of rationales allowing faster discovery of potent and selective AMPs under specific conditions,[^8^, ^9^, ^10^] but those methods are still relying on experimental data to ascertain the structure to function relationship of AMPs and membranes interactions. Thus and in parallel, there is a primary impetus for developing analytical tools able to provide detailed information on the structure of AMPs, their molecular specificities, as well as directly and rapidly probe the nature and the extent of their interactions in bio‐applicable environments.[^7^, ^11^, ^12^] Complementary methods have to be applied to gather deeper insights onto these systems.[^13^, ^14^]

Non‐invasive optical methods such as circular dichroism (CD) spectroscopy or vibrational spectroscopy using infrared radiation (IR) are promising techniques for meeting these goals. In particular, and unlike other methods, Fourier transform infrared (FTIR) spectroscopy can provide detailed information on both primary and secondary structure of proteins and polypeptides,^[15]^ as well as their relative orientation in relevant membrane environments (liposomes,[^2^, ^16^] bilayers^[5]^) of various interaction patterns.[^17^, ^18^] Supplemented with 2D correlative analysis, FTIR becomes a powerful tool to put into evidence the potential interdependencies and interactions between peptides and their (native) molecular environments. Complementary to the mid‐IR region (MIR), the use of the low frequency region, far‐FTIR (FIR)^[19]^ or terahertz (THz)‐time domain^[20]^ is intrinsically attractive as it discriminates vibrational modes involving inter‐ and intramolecular hydrogen bonding^[21]^ which, due to their collective nature, are highly sensitive to the conformational state of the molecule.^[22]^ Such a capability can then provide a unique fingerprint for a specific molecular arrangement. Hydrogen bonds indeed play a critical role in supporting different inter‐ and intramolecular events ranging from charge shifting, molecular stabilisation, affinity and recognition including those in bio‐relevant systems which can induce particular folding patterns.^[23]^

As temperature (T) has a large effect on hydrogen bonding and reduction of thermal vibrations, the development of the FIR signatures with changing temperature may not only reveal increases (or decreases) of the number of hydrogen bonds, but also fine structural re‐organisations to the point of clarifying the role of intermolecular forces in conformational transitions,[^24^, ^25^] which further discriminates one system from another.

This study tackles the non‐invasive chemical identification of biologically relevant thin films prepared onto HDPE substrate foils in the FIR spectral region from 400 to 40 cm^−1^. Static T‐dependent Synchrotron Radiation (SR)‐based FIR spectroscopy in the 298–10 K range was applied to model peptide templates (cationic AMP and anionic non‐AMP used as negative control) and reconstituted mammalian and microbial model membranes, before and after contact. Together with 2D correlation tools,[^26^, ^27^] FIR reveals specific molecular patterns of peptide‐lipid membrane interactions of direct relevance to membrane‐mediated antimicrobial function.^[25]^

## Results and Discussion

### Antimicrobial Peptides’ Specifics

To probe AMP folding by synchrotron radiation far‐IR spectroscopy, two model peptides were prepared as reported elsewhere.^[28]^ The modes of action for these peptides have been comprehensively characterised using low‐ and high‐resolution spectroscopy and imaging tools, as well as biological assays.[^28^, ^29^, ^30^] The peptides are an archetypal α‐helical cationic AMP (KARLA), which was shown to have antimicrobial activities comparable to those of other AMPs and antibiotics, and a non‐AMP anionic peptide (QAELA), which was used as a negative control as it lacks any biological activity.^[29]^ The two peptides have the same helical propensity, but different folding responsiveness upon binding to membranes (Table 1).

**Table 1:** Overview of the peptides used in the study. With Q: Glutamine, A: Alanine, E: Glutamic Acid, L: Leucine, K: Lysine, R: Arginine. MS [M+H]^+^: mass spectrometry decomposition value of the metastable peptide [M+H]^+^ ion, m/z: mass to charge ratio, am: amide group NH_2_.

Name	Template	Sequence	MS [M+H]^+^,	m/z
			(calc.)	(found)
QAELA	non‐functional negative control	QAELAQLEAQLYELQAELAEL–am	2372.6	2372.9
KARLA	functional	KARLAKLRARLYRLKARLARL–am	2536.2	2537.4

### T‐Dependent FIR Characterisation of Peptide Template Films

All FIR studies were conducted on thin films in order to probe molecular changes of the hydrogen, oxygen, nitrogen as well as carbon backbone bonds as a function of structural and conformation preferences under external stimuli, here temperature. Investigations on sample pellets instead do not provide exact band profiles in presence of an embedding medium (cf. the Supporting Information (SI) Figure S3).

A near‐native sample preparation that maintains conditions as close as possible to the physiological conditions for the peptide and for the model membranes has obvious advantages as it preserves at best the “native” structural contributions of both components’ reactivity. The incubation of the peptides with the model membranes was therefore realised in “native aqueous state”. This method allowed to ascertain that the peptide protective solvation shell is maintained, that secondary structure modification and higher order structure formation are largely avoided, and to maintain full reactivity of the system.[^31^, ^32^] FIR investigations were conducted on the subsequently dried films. Systematic study and 2D correlation within the same experimental conditions were undertaken, and derived observations were found to be accurate and highly reproducible, as demonstrated by T‐runs reversibility curves (cf. SI, Figure S4). These FIR investigations on dried films equally concur with observations in the complementary MIR range performed under hydrated conditions (cf. SI, Figure S9), and no significant changes of conformation or orientation were seen between analyses performed under liquid and dried conditions. Conclusions issued from FIR and MIR measurements (Figure S9, SI), NMR and CD^[29]^ were found to be consistent.

Interestingly in the FIR, a continuum is found at about 400 to 0 cm^−1^, where the exact position and broadness of the bands depend on the polarisability of the hydrogen bonding features,[^33^, ^34^] i. e. on collective motions of the carbon backbone and hydrogen bonds.

**Figure 1:**
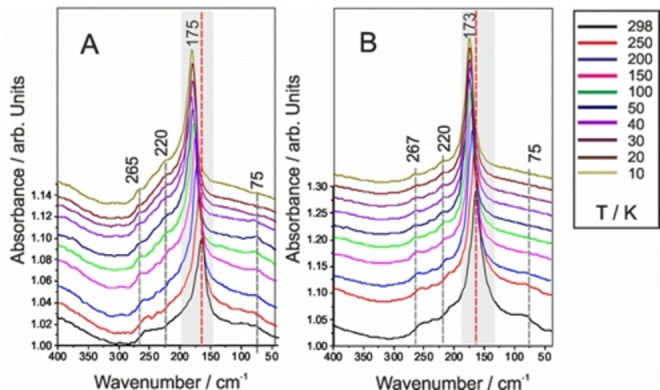
FIR spectra of A) anionic QAELA and B) cationic KARLA peptide films. Spectra were recorded between 400–40 cm^−1^ and in the T‐range from 298 to 10 K. The collective mode, e. g. Amide VII domain, is highlighted in gray and gray/red dashed lines evidence relevant T‐dependent spectral evolutions.

The most prominent spectral feature that QAELA (non‐AMP) and KARLA (AMP) (Figure 1) display in common is a primary band in the region from 180 to 160 cm^−1^ (s), the position of which is dependent on the molecular structure of the respective peptide sequence at a certain temperature (domains highlighted in gray). This band is identified as structural collective modes (torsion −CN and −CO_2_H bonds mainly, with an ensemble of hydrogen bond vibrational modes) that are associated with the peptide backbone, which can be observed in alanine‐rich peptides.^[32]^ These modes include in particular the Amide VII mode, which is a composition of N−H out of plane, C−N torsion, and C=O out of plane bends (see Table 2), as previous investigations on other peptides have reported.[^35^, ^36^] More specifically, the strong mode at about 170 to 175 cm^−1^ (s) can be assigned to torsion modes (−CO_2_H) of alanine residues in the peptide,^[21]^ together with a torsion mode that derives from the C−N bond. Other series of modes between 265 (w) and 215 cm^−1^ (w) are present, which most probably originate from the other amino‐acids (e. g. glutamic acid and lysine), and typical of torsion (220, 240 cm^−1^ (w)) and rotational (220 cm^−1^ (w)) modes. Due to the cooling‐down process, this Amide VII mode is shifted to shorter wavelengths. Lower temperatures are leading to a reduction of thermal vibrations, a generation of higher‐ordered structures prompted by T‐induced phase transitions, and an increase in strength of the hydrogen bonds.^[36]^ These observations are in agreement with previous investigations on proteins, where a liquid‐glass transition around 150–180 K could be observed, together with a linear increase of the signals.^[24]^ A shoulder at ca. 250 cm^−1^ (w) can be observed for both peptides; it is linked primary to various out of plane bends of the peptides backbone. Another important band is located around 100 cm^−1^ (br), and is associated to the intrinsic mode of hydrogen bond, accordingly decreasing in intensity with temperature, presumably reflecting the re‐organisation of internal water molecules.^[22]^ Accompanying this, a complicated spectral domain, i. e. below 100 cm^−1^, also presents information related to the hydration or solvation shell of the peptide, both of which have been demonstrated to be crucial for proteins.^[37]^ This domain separates the absorbance induced by bulk water from the ones of water molecules forming the solvation shell.^[29]^ In addition, for KARLA especially, one can observe further modes located between 57 and 40 cm^−1^ (w) referring to intra‐chain hydrogen bonds, known for forming secondary structural elements such as α‐helices and β‐folds structures. This is in accordance with MIR investigations undertaken on the cationic peptide KARLA in solution, which revealed mainly an unfolded conformation with some important α‐helical, and to a lower extent, some β‐turn contributions (cf. SI, Figure S9) expected conformational preferences for this peptide. Hence complementary vibrational information can be obtained at different levels of the molecule by switching from MIR to FIR, where conformation states can be gathered from the carbon or nitrogen bonds backbone and sidechains for instance, and from the hydrogen bonds’ intra‐chain, respectively.

**Table 2:** FIR modes of the peptide films and their corresponding assignments. δ: deformation, ṽ: stretching, τ: torsion.

Mode [cm^−1^]	Tentative assignments	Refs.
40 (w)	intra and intermolecular H bonding signature	[38–40]
57–83 (w)	H bonds, water bending modes	[41,42]
94–110 (br)	intrinsic mode of H bond	[43]
115 (br)	intramolecular N−H…O, H bond associated with Amide VII	[44]
132 (w)	shoulder related to Amide VII, H bond	[45]
164–180 (s)	Amide VII, −N−H *out‐of‐plane* bend, τ(−C−N), −C=O *out‐of‐plane* bend, τ(−CO_2_H), H vibrational modes associated with backbone	[32,36,46,47]
200–220 (w)	N−H *out‐of‐plane*, τ(−NH), τ(−CH_2_), C bonds	[36]
240 (w)	τ(−CO), τ(−NH), τ(‐C−CH_3_)	[41]
250–275 (w)	δ(−C−N−C), δ(−C−C−N), −C=O *out‐of‐plane* bend	[48]
300 (w)	τ(−C−C)	[49]
341 (m)	τ(−C−C−N)	[49]
355 (m)	Skeletal deformation of the CH_2_−CO−NH−CH_2_ groups, δ(−C−C−N), C=O *in plane* bend, ṽ(−CN)	[50]
380 (w)	τ(−C−C−N), related to α helices	[44,51]

For QAELA, a stronger T‐dependency can be observed (especially on the Amide VII mode), whereas for KARLA, while T‐dependent changes also occur, they are of lesser decrease/increase at the respective temperature. This indicates a lower reactivity of KARLA towards temperature, with a molecular organisation less prone to thermal instabilities, via either fewer or structure‐stabilised internal water clusters, or via stronger intramolecular bonds in general, which may reflect a limitation in structural adaptability (change in conformation).

### 2D Correlation Analysis on Peptide Sample Films

The 2D correlations analyses were conducted in the entire T‐range (298–10 K), and their maps are therefore the reflection of the average behaviour of the most prominent spectral changes occurring to the samples upon cooling. These 2D correlations however do not exclude specific evolutions or multiple competing molecular groups’ dynamics that may take place at specific temperatures.

**Figure 2:**
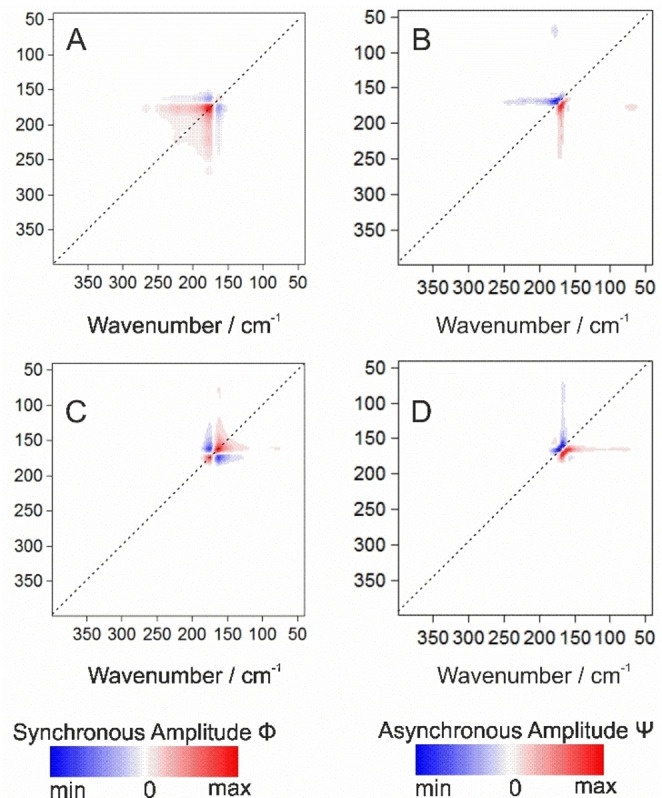
2D correlation analysis of T‐dependency (298–10 K range) in the 400–40 cm^−1^ spectral domain of QAELA (non‐AMP) and KARLA (AMP): A) synchronous and B) asynchronous maps of QAELA; C) synchronous and D) asynchronous maps of KARLA.

For synchronous results Figures 2A and 2 C, one can notice two prominent auto‐peaks at 175 and 165 i. e., patterns on the dashed diagonal, and two cross‐peaks at 175 and 165 out of the dashed diagonal. These describe changes in signal intensity induced by a change in temperature for the same and for different spectral ranges, respectively. The strongest changes in intensity can be observed at ca. 175 cm^−1^ (s) for collective modes consisting of the Amide VII and others structural and H bonds vibrational modes from peptide backbone.^[32]^ Its intensity is increasing (red peaks, positive peak amplitudes), while the temperature is decreasing. This indicates simultaneous changes in the spectral intensity in the same direction which occurs for all the bands composing specifically this spectral region, i. e. monotonous bands evolution. One can observe however an asymmetry in the shape of the auto‐peaks and lobes which point towards higher (QAELA) or lower (KARLA) wavenumbers. This demonstrates the presence of overlapped contributions, as well as peaks shifting towards each other, which grow in the same direction as the peptide backbone mode and of the Amide VII in particular. For QAELA, these elongated “streaks” are linking the Amide VII mode T‐dependency to the main bands of the peptide backbones and amino‐acids {e. g. at coordinates [ca. (266,177), (240,177), (221,177)]}, whereas for KARLA it is correlated to intra‐ and intermolecular H bonds [e. g. (130,160), (125,160)]. Additionally, two main cross‐peaks appear (blue, negative peaks amplitudes), indicating T‐dependent changes for different wavenumber regions which evolve in opposite directions. For instance, in Figures 2A and 2 C, this contrary intensity pattern can be noticed at the inflection points at ca. (157,175) of the Amide VII mode. These cross‐peaks, also of elongated forms, imply that the Amide VII bands contributions at 165 for QAELA and 175 for KARLA are decreasing while the content of the other molecular groups, peptide backbone and H bonds, respectively, are increasing. These interpretations are confirmed by the presence, for QAELA, of another positive auto‐peak at 221 (backbone torsion band) and a separated positive cross‐peak at (266,177) related to the peptide backbone modifications, and for KARLA, of a cross‐peak at (162,79) reflecting water content increases. Other than mirroring the spectral variability (positive/negative slopes) in these regions, which points out the T‐induced modifications on the molecular conformation of the peptides’ backbone components (e. g. Amide VII), this demonstrates the capacity of 2D correlation analysis to deconvolve complex bands resulting of many contributions, and to highlight band co‐dependencies.

The T‐dependent spectral output can provide further insight when analysing the asynchronous spectrum which, via its cross‐peaks, underlines the sequential changes of the spectral intensities. Both in Figure 2B and 2D, the asynchronous 2D cross‐peak at (177,169) is negative (blue) and the following asynchronous 2D cross‐peak at (169,177) is positive (red), while the intensity in the corresponding peak of the synchronous spectrum (Figures 2A, 2C) is negative. Interestingly, for QAELA the band at 177 cm^−1^ (s) shows a correlation with the 245–177 domain through its elongated form, and also shares a cross‐peak of opposite sign at (71,177). KARLA instead presents a correlation of the 165 cm^−1^ (s) band with the 144–79 domains, and displays another cross‐peak at (177,155). This conforms the results of the synchronous plots and delineates more precisely the various bands correlations which, in the case of asynchronous plots, are moving successively in time. Moreover, applying the rules determining the sequence of events,^[52]^ we can deduce that the T‐change at 177 cm^−1^ (s) precedes slightly the T‐change taking place at 169 cm^−1^ (s). This may reflect backbone (Amide VII) conformational changes, or alanine residues modifications.

Basically, both synchronous and asynchronous spectra reveal that the changes in absorbance most often occur simultaneously for all the molecular groups. Thus, no change issued from one specific molecular component is triggering other secondary changes, distinct from those which are T‐dependent.

### T‐Dependent FIR Spectroscopic Investigations of Model Membranes and 2D Correlation

**Figure 3:**
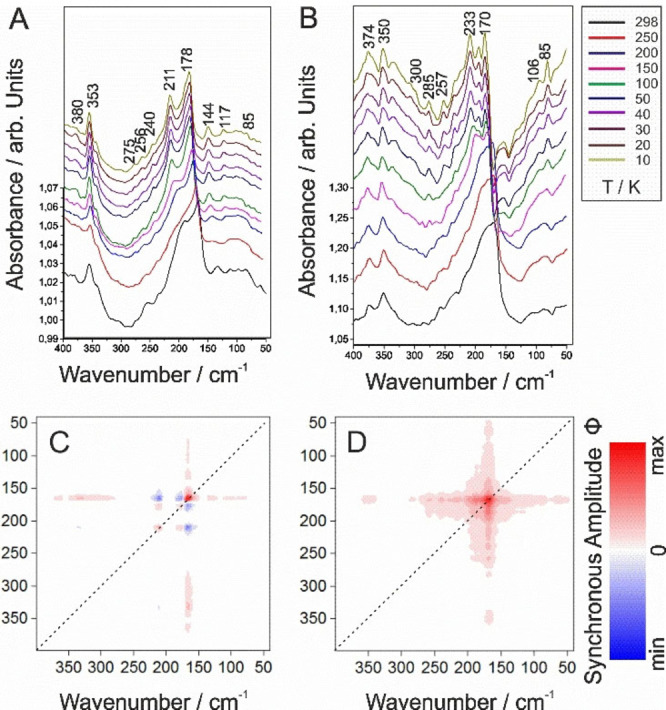
FIR spectra in the 400–40 cm^−1^ spectral range of A) ZUV and B) AUV membranes. Corresponding synchronous 2D correlation plots are given in below, respectively, for C) neutral ZUV and D) anionic AUV membranes, in the 298–10 K T‐range.

Anionic unilamellar vesicles (AUVs) and zwitterionic unilamellar vesicles (ZUVs,) were prepared to mimic microbial and mammalian phospholipid membranes, respectively. ZUVs were assembled from 1‐palmitoyl‐2‐oleoyl‐sn‐glycero‐3‐phosphocholine lipids (POPC), while AUVs were assembled from POPC with 1‐hexadecanoyl‐2‐(9Z‐octadecenoyl)‐sn‐glycero‐3‐phospho‐(1’‐rac‐glycerol) (POPG) at a 3 : 1 molar ratio. These liposomes both contain hydrophobic chains and hydrophilic head groups, similarly to natural cellular membranes. Their FIR spectra are shown in Figure 3, and further details related to band assignments can be found in Table S1 (cf. SI).[^25^, ^53^]

Both reconstituted membranes are characterised by two intense signatures at about 178 and 170 cm^−1^ and 211 and 233 cm^−1^, for the neutral ZUVs and anionic AUVs, respectively. As described in the SI section 2.6.1, these bands are attributed to intermolecular hydrogen bonding structure and to the torsional mode of the hydrocarbon chain, and to other torsional modes of the hydrocarbon chain, respectively. With T, those bands are shifting and additional contributions are arising, which all point out towards important modifications of the liposome backbone and modification of charges positioning.

In addition, these neutral ZUV and anionic AUV membranes (Figures 3A, 3B) both deliver a broad absorption pattern between 150 (s) and 40 cm^−1^ (w), a spectral range attributed to molecular breathing and to intermolecular hydrogen‐bonding structures within the phospholipid layers.^[25]^ Especially, the band related to intrinsic H modes around 85 cm^−1^ (w) is found to decrease with temperature and shifts towards 95 cm^−1^ (w). This is explained by a re‐organisation of internal water molecules which modify the interaction between the liposomes and the water molecules,^[25]^ and in turn depends on the composition of the lipids, as well as on the head groups involved in the liposomes’ reactivity. Further, torsion modes referring to hydrocarbon chains in the spectral range between 300 and 180 cm^−1^ (w) can be detected. T‐stable modes common to both reconstituted phospholipid membranes are located between 380 (w) and 350 cm^−1^ (m) and refer to the choline groups of the liposome formulations. Most of the investigated spectral domain reveals the presence of the same molecular groups for both peptides, which is in accordance with the groups composing both ZUV and AUV membranes (cf. SI, Table S1). Evident differences in the bands’ intensity ratios are however noticeable between both membrane types, as well as some spectral specificities. In particular, the connectivity band (domain below 280 cm^−1^) displays a multi‐component structures, composed primary by inter‐layer hydrogen bounded water bridges arising from the head groups of choline, phosphocholine or other polar groups,^[54]^ as well as torsional modes of the hydrocarbon chains (cf. SI, Section 2.6.1), and reflects significant differences in the ZUVs and AUVs respective molecular structures. These shifts in the bands positions of both ZUV and AUV membranes may be remotely linked to the overall charge positioning offered by these molecules within or at the surface of the liposome, and differences in solvation of polar or head groups, i. e. linked to their binding affinities.

2D correlation analysis (Figure 3) efficiently allows for a rapid evaluation of the T‐effect onto the membranes and are mirroring the spectral analysis while highlighting the main results. ZUV membranes’ synchronous map (Figure 3C) shows 3 auto‐peaks (165 (s), 175 (s), and 210 cm^−1^ (m,s)), which readily identify the main groups involved in the T‐changes upon cooling, i. e. the internal hydrogen bonding structures, and the torsional modes of the hydrocarbon chain, respectively, both linked to the head group of phosphocholine. These point out towards a modification of the hydrophilic backbone of the liposomes, as expected. The most important change occurs at 165 cm^−1^ (s), with an increase in the spectral intensity; the two other auto‐peaks increasing equally. Cross‐peaks are observed within these groups’ wavelengths at (210,165), (210,177) (175,165) but also with others, e. g. at (165,133) and (165,366), and in form of elongated lines [i. e. (165,177–120) and (165,275–335)]. This indicates that the main variation in intensity at 165 cm^−1^ (s) (internal H bonding and torsion of the hydrocarbon chains linked to phosphocholine groups, the later confirmed by a co‐dependency of the molecular vibration at 366 cm^−1^ (m,w)), is accompanied by intensity changes of the same sign (red) of the hydrocarbon chains and, to a lesser extent but of the same sign, by modification of the various types of H bonds. These changes are also linked to inverse variations (blue) of the bands at 175 (s) and 210 cm^−1^ (m), which reveal specific torsional modes of the backbone chain as well as a modification of the phosphocholine conformation.

For AUVs (Figure 3D), a prominent cross‐pattern, centred around the auto‐peak at 165 cm^−1^ (s) is characteristic of band intensities which are synchronously evolving in the same direction (red) within the entire T‐range considered. Extended lobes (and a probable auto‐peak at 190 cm^−1^ (s)) are revealing strong interactions and overlapped contributions between the various molecular groups related to the backbone chains as well as to the H‐bonds and water modes. Despite a similarity of evolution with neutral ZUV membranes, the molecular changes are slightly different, as visible from the higher co‐dependence in the changes linked to the phosphocholine groups with the intermolecular H bonds or intrinsic H bonds (150–80 cm^−1^ (w) domain), as well as from different deformation modes of the backbone chain (torsional and deformation), which reflect a specific molecular sensitivity of the considered liposome towards T.

Asynchronous results are presented and discussed in the SI (Section 2.6.2, Figure S5).

### FIR study of AMP‐Model Membrane Interactions

The systematic studies performed to this end helped acsribe the spectral features related to T‐dependent structural changes in the peptide or in the membrane model sample films, considered separately. This allowed us to probe the propensity of the archetypal peptide sequences to effectively interact, or not, with the model membranes. All amino acids in the peptides used have high helical propensities. This makes the two peptides comparably helical, but with only one exhibiting selective membrane binding.^[55]^ Electrostatic interactions between cationic KARLA (AMP) and anionic membranes (AUVs) induce and stabilise helix formation whilst further stabilisation via water‐amide interactions occur, which allow the antimicrobial peptide to fold and assemble in the membranes in a certain orientation.^[56]^ The electrostatic interactions, depending on charges balance and repartition, specify the overall affinity of the AMP to microbial membranes, while this process is not favourable in our control cases (cf. SI, Sections 2.7.2.2–2.7.2.4). It has indeed been proposed that the ratio of cationic residues in peptides can be tailored to achieve an optimal charge stoichiometry between peptides and lipids leading to enhanced peptide affinity to anionic membranes. Such optimisation of peptide lipid polar groups can be used as a design strategy for more effective antiviral and antimicrobial drugs.^[12]^

### Control Results

**Figure 4:**
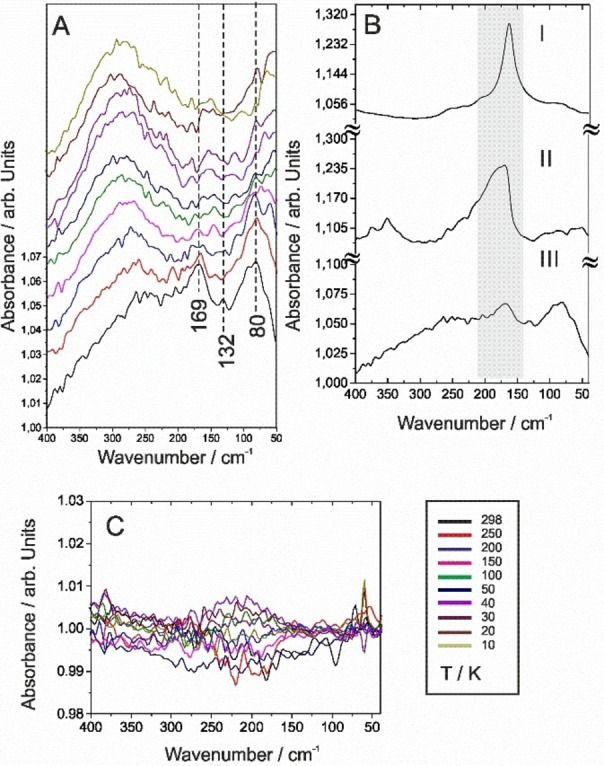
A) FIR spectra of AUV membranes with KARLA peptide. B) FIR signatures of (I) KARLA, (II) AUV membranes, and (III) AUV membranes with KARLA at T=298 K, and of C) AUV microbial membranes with QAELA control peptide.

No apparent interactions were observed between the negative control template QAELA and the microbial model membranes (Figure 4C, also cf. SI, section 2.7.3). No appreacible signal which could reveal detectable modes specific to QAELA in the 200–150 cm^−1^ region could be ascertained. In addition, no re‐organisation or modification of H‐bonds or water domains can be resolved. Similar behaviours observed for both QAELA and KARLA in zwitterionic membranes indicate that the two peptides did not bind to the membranes and remained remained unfolded and repulsed in presence of the ZUVs (cf. SI, Section 2.7.1, Figures S6–S7), which are mostly neutral.

The results and discussion section is focused on the interactions between the cationic AMP (KARLA) and the bacterial model membrane (AUVs). All control experiments, including 2D correlation data analysis, are detailed and discussed in the SI (Section 2.7.2, Figures S7‐S8).

Figures 4A and 4B(III) show important T‐dependent alterations of the AUVs spectra in the presence of KARLA upon cooling. In sum, the following features can be observed: i) a salient modification of the band at ca. 170 cm^−1^ (s), which results from a convolution between the Amide VII mode of KARLA and the hydrocarbon chain groups and H bonds with the phosphogroups of AUVs; ii) an increase of the band at ca. 130 cm^−1^ (m), demonstrating further changes in conformation of the Amide VII band together with a modification of the H‐bond of the AMP‐AUVs system; iii) the appearance of a prominent shoulder at 80 cm^−1^ (br) due to extensive reorganisation of water molecules and bending modes; and iv) an increase of the background level. In addition, the FWHM of the mode at ca. 170 cm^−1^ (m) (30 cm^−1^) resembles the one observed in the KARLA spectrum (compare Figure 4BIII with Figure 4BI). The slight shift of the Amide VII band towards lower wavelenghts, was also demonstrated to be a marker of the peptide or protein binding.^[19]^ The overall modification of the connectivity band, and in particular the modifications linked to the conformational and backbone changes, and those linked to the inter‐chains hydrogen bonds, i. e. hydrocarbon chains, together with all the above‐mentioned molecular features indicate the embedding of KARLA in the negatively charged membranes.

In detail, the T‐dependent series (Figure 4A) reveals three main spectral characteristics in the AMP‐AUVs interactions: the band intensity at 80 cm^−1^ (br) decreases with T while the other features below 100 cm^−1^ moderately increase. This is consistent with an extensive re‐organisation of internal water molecules and of the water bending mode occurring in the system, and providing evidence for disorder in the membranes due to interactions with the peptide. The mode at 170 cm^−1^ (s) decreases as the one at ca. 130 cm^−1^ (m), both mostly related to the peptide backbone Amide VII and other associated intrinsic H‐bonds, while a small increase of the 210–235 cm^−1^ (m) (linked to various amino‐acids) occurs, which is consistent with the modification observed for KARLA alone. Finally, the appearance of a wide band at ca. 275 cm^−1^ (w) slightly shifting towards lower wavelengths with T, the lack of defined bands related to hydrocarbon chains, and the overall augmentation of the background demonstrates complete disorder in the glycerol backbone of the phospholipid membrane (and its probable rupture), as well as in liposomes side‐chains, as a result of interactions with KARLA. Typical of AMPs, KARLA comprising polar neutral, cationic and hydrophobic residues arrange into amphipathic helices in anionic membranes, but adopt majoritarily random coil (unfold) conformations in solution or in the presence of neutral membranes, as confirmed by FIR (Figure 1) and MIR (cf. SI, Figure S9). The electrostatic interactions between the AMP and anionic membranes (AUVs) mediate the transition between random coil and helical conformations with peptide helices intercalating in the glycerol headgroup regions of the lipid bilayers. The associated changes in the Amide band are detectable in the spectra (at 170 (s), 130 cm^−1^ (w)). Further, the changes in the domain below 100 cm^−1^ suggest a disruption of hydrogen bonding structures, induced by electrostatic interactions of the AMP with AUVs membrane components (e. g. protonation of amino‐acid residues), and their interplay with hydrophobic interactions at the lipid interface. The conformational transition into α‐helices determines the insertion and migration of AMPs in membranes correlating with antibacterial activity,^[57]^ mechanism that is confirmed by the obtained FIR spectra (Figure 4‐BIII). Indeed, migrating AMPs can cooperatively assemble into developing pores and lesions[^2^, ^58^] or disrupt membranes in a carpet‐like manner^[59]^ or by inducing fractal ruptures.^[60]^ Of particular interest, within the changes in the connectivity band, are the modifications linked to backbone chains and to interlayer‐hydrogen bounded structures (e. g. water bridges). The degree of hydration of these environments was postulated to be an important component to hydration attraction forces in phospholipids, via solubility‐diffusion mechanisms,^[54]^ while the phononic motions of hydrocarbon chains, via interlayer hydrogen‐bounded water bridges were recently demonstrated to mediate the passive transport of molecules such as peptides across membranes.[^61^, ^62^]

In marked contrast, QAELA did not fold in either AUVs or ZUVs,^[28]^ as demonstrated in the SI (Section 2.7.2, Figures S7–S8). NMR spectroscopy provided further detail as to the orientation of KARLA in AUVs and disordering effects it caused in the phospholipid bilayers of the membranes. The peptide was shown to tilt in the bilayers and perturbed their upper leaflets, decreasing mobility of phospholipid headgroups.^[28]^

### Synchronous 2D Auto‐Correlation Analysis of KARLA‐AUVs System

**Figure 5:**
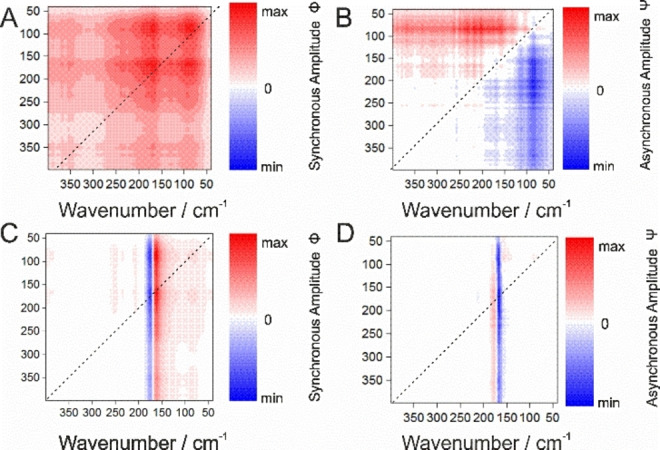
A,C) Synchronous and asynchronous (right) 2D correlation maps of AUV membranes with KARLA (auto‐correlation) and B,D) KARLA (A(ν_1_)) versus AUV membranes with KARLA (A(ν_2_)) (cross‐correlation).

The 2D synchronous auto‐correlation analysis of KARLA‐AUVs system (Figure 5A) indicates that the T‐changes occurring upon cooling lead to monotonous band evolutions, i. e. in the same direction (red) as their spectral intensity diminishes for all of them. Intense auto‐peaks at ca. 80 and 170 cm^−1^, accompanied by a weaker one at ca. 360 cm^−1^, confirm the effective binding to and disruption of the AUV anionic membranes by KARLA. They indicate (i) changes in structured water in peptide‐membrane systems, ii) the Amide VII band (and related collective backbone contributions) of KARLA convolved with the hydrocarbon chains supporting the phosphocholine groups of the AUVs (e. g. aliphatic chains) and their relative conformational changes, and iii) the deformation and probable collapse of the AUVs hydrocarbon backbone, introducing disordered phases, with all the connectivity band domains changed.

The cross‐peaks (other than those issued from combination with the auto‐peaks) at 350 (w,sh) with features at 80 (w,br), 130 (m) and 190 cm^−1^ (s); and at 235 (br,w) with features at 80 (br) and 170 cm^−1^ (s) further demonstrate the changes taking place in the backbone chains and in the system water (H‐ and CN‐bonds), and which arises from membrane deformation to potential disrupture of the ordered phases’ structural bonds, as gauged by the diminution in global spectral intensity. The increase of the noise level, as well as the importance of the molecular changes and diminution of the ordered structures is consistent with that KARLA induces substantial disorder in AUV membranes, in agreement with the FIR results (Figure 4).

### Synchronous 2D Cross‐Correlation Analysis KARLA versus KARLA and AUVs

By plotting 2D cross‐correlation results, i. e. KARLA versus KARLA and with anionic AUV membranes (Figure 5C), the spectral domain corresponding principally to the AMP responses when bound in the AUV membranes, and with respect to the T‐dependency is enhanced, highlighting subtler molecular changes. This plot reveals the main intensity changes in KARLA within the AUVs and is used to deconvolute the spectral contributions (and co‐dependencies) of both KARLA and AUVs.

As anticipated, the band at 165 cm^−1^ (s) (red) presents a spectral intensity change moving in opposite to the feature at 175 cm^−1^ (s) (blue), a gap clearly separating the two stripes. This denotes a change in the peptide conformation, linked to all (or most) molecular species evolving synchronously and mostly monotonously with the Amide VII band. A small auto‐peak is discernible at 82 cm^−1^ (br,s), which demonstrates a simultaneous modification of the water domain (and inclusive of structured water in the KARLA‐AUVs system). The co‐dependency with the other molecular groups of the system is indicated by a long series of streak lines in the plot: at 261 (w), 241 (br,w), 210 (br,w) and 82 cm^−1^ (br,s). The first three denote a modification of the hydrocarbon chains, which is codependent of the changes taking place for the H‐bonds (stripes from ca. 70 to 210), while the one at 82 cm^−1^ (br,s) reveals a global perturbation of the molecular system, specifically due to changes in all related H‐bonds. The cross‐correlation analysis univocally highlights a conformation change in the AMP upon binding to AUV membranes, as well as the protonation of the amino‐acid side chains.

### Asynchronous 2D Auto‐ and Cross‐Correlation Analyses

To get a better insight into the sequential changes of the spectral intensities (out of phase events), 2D asynchronous auto‐and cross correlation analyses of KARLA‐AUVs system were also performed.

The asynchronous 2D auto‐correlation map (Figure 5B) clearly indicates contributions from overlapping bands resolved in the synchronous plots. In particular, it emphasises that the T‐changes in the Amide II collective band at 170 cm^−1^ (s) trigger all other modifications, and especially those at 83 cm^−1^ (br,s), i. e. of the water domains. However, the most intense feature is the streak line at 83 cm^−1^ (br,s), which in turn correlates with further changes taking place at 170 cm^−1^ (s), as well as those occurring to the various structures of the molecular backbone. Its intensity and sign tend to indicate that, after the primary changes of peptide conformation, predominantly water and intermolecular H‐bond changes precede any other changes (e. g. specific to the liposome backbone) happening in the AMP‐AUVs system.^[63]^ It must be noted that at this point the noise contributions become apparent in the spectra. As asynchronous plots are sensitive to the impact these contributions may have on the results, the analysis was not pushed further.

The cross‐asynchronous plot of KARLA versus KARLA with AUVs (Figure 5D) only allows to generally draw conclusions about the codependencies of multiple bands and out‐of‐phase events, but does not allow establishing the sequence of the spectral events in contrast to what was achieved with the auto‐asynchronous analysis. The bands at 165 (s) and 175 cm^−1^ (s) develop synchronously, indicating a change in conformation. The small elongated cross‐peak at ca. (210,170) related to deformations (e. g. torsions mainly) of the backbone (e. g. NH‐bonds), increasing together with the band at 175 cm^−1^ (s), confirms the conformational changes. Already visible in Figure 4A and 5 A, these T‐changes are affecting all molecular groups present in the AMP‐AUVs system, and especially the conectivity band of the membrane. The cross asynchronous analysis confirms that KARLA induces the changes in the system, as all spectral modifications are subsequent to the AMP's conformational changes triggered by the contact with the AUVs. These 2D correlation asynchronous maps reveal the multi‐steps processes involving the codependencies of specific molecular groups in a timely resolved manner, which occurred as a result of AMP‐AUVs interactions, and which support membrane‐mediated folding bioactivity.

Summarising, 2D correlation analysis of peptide versus the peptide‐model membrane complexes is an appropriate tool to reveal molecular variations as a function of T. It demonstrates that it can readily discriminate the level of biophysical‐chemical interactions, and can gather information about co‐dependencies of molecular groups quickly and independently. No quantitative information however is available on the real amount of interaction. These findings are complementary and consistent to the results of earlier studies on the peptides by CD and NMR spectroscopy.[^28^, ^29^, ^64^] Specifically, CD spectra were found to be strongly helical for KARLA in AUVs, whereas in ZUVs the spectra were characteristic of random coil conformations.^[28]^

### Discussion and Proposed Mechanisms of Interaction

KARLA (AMP) interactions with anionic AUVs membranes prove to be balanced between electrostatic interactions, hydrophobicity and entropic potentials which are major driving forces for binding to cell.^[61]^ In this process, cationic moieties are involved in the initial interactions with the liposome, while the hydrophobic face of the folded helix mediates peptide insertion and intercalation in the hydrophobic bilayer interface. During this intercalation process, KARLA introduces hydration shells that promote the re‐organisation/re‐orientation of the affected lipid domains in the membrane and help to further diminish its structural order.^[29]^ This is exactly what is reflected in our 2D correlation asynchronous maps, which reveal that after a change in conformation of the peptide, the water domains and then all molecular groups linked to the membrane are affected. Other studies have shown that charge disruptions in the membranes result in the loss of molecular (lipid) components, and other complex phenomena, which can influence the selectivity of AMP binding.[^9^, ^11^, ^65^] Thus, in the study of AMP‐anionic membranes systems, 2D correlation analyses complement FIR spectra to prove the destabilisation of the system via a diminution of its structural groups, and its disorder, indicating the disruption of the liposomes. This is accompanied by subsequent aggregation events such as lipid clustering and micellisation. Indeed, accumulation of AMPs in the outer part of the leaflet of the negatively charged bacterial membranes might have lead to enhanced interfacial tension disturbing or disrupting the liposomes. With an exchange rate set to one hour, the interactions between the AMP and the microbial model membrane were appreciable, which is consistent with the results obtained using giant unilamellar vesicles of the same lipid type.^[29]^ The kinetics of interactions can considerably vary^[66]^ for different peptides, as those depend on the peptide physiological state, the binding of amino‐acid residues or ligands, or even on their interactions with membranes, depending on the conditions used.

## Conclusions

The Far‐infrared to THz spectral window is often deemed not to be as much information‐rich, compared to the conventional mid‐infrared spectral window traditionally used for detection of molecular vibrations and functional groups, or to NMR, which allows to identify an exact peptide sequence. But FIR access to vibrational modes involving T‐dependent inter‐ and intramolecular hydrogen bonding unlocks new and unique fingerprints for a specific molecular arrangement. Here, FIR has been successfully applied to the study of non‐AMPs and AMPs, reconstituted phospholipid membranes, and to the mechanistic elucidation of AMPs in such membranes as a function of temperature. Each peptide template exhibits a unique spectral fingerprint which notably revealed specific patterns of intra‐chain hydrogen bonding, improving thus the understanding of AMPs structure‐activity relationships. T‐dependent FIR‐analysis, supported by 2D correlative tools, has indicated that spectral interactions between selected AMPs and reconstituted phospholipid membranes, underline the molecular structural organisation specificities, and can be directly related to antimicrobial mechanism and ultimately function. Asynchronous 2D correlation analysis, in particular, expanded the FIR potential by retrieving the sequence of reorganisation of the main molecular groups which participate to the interactions. This new approach was at the core of our findings.

As expected, no interaction was observed between the membrane‐inert QAELA and neutral (ZUVs) and anionic (AUVs) model membranes. In contrast, spectral features assignable to “reaction and bonding”, and indicating a strong peptide‐lipid interaction as for KARLA and the microbial anionic AUV membranes, confirmed that antimicrobial activity is mediated by membrane binding.

Admittedly, all aspects pertaining to peptide interactions with membranes cannot be captured in one study. Nonetheless FIR provides a straightforward probe of the main steps in antimicrobial peptide‐lipid interactions in microbial membranes, that can be correlated with folding‐induced mechanism of antimicrobial peptides. These T‐dependent FIR‐studies provide exploratory insights into the molecular organisation of peptides, artificial membranes and their interactions, whereas the 2D correlations prove to readily discriminate between different spectral features and to relate to specific molecular interactions. This approach highlights in particular the importance of cooperativity in hydrogen bounded structures in the liposomes, disrupted by and supporting the incorporation of the peptide (in our case, the AMP).

The present results demonstrate the potential of FIR in advancing the development of novel antimicrobial agents by accessing a peptide molecular structure while probing its potentially (quantifiable) response and bioactivity, and thus, by ultimately providing molecular‐scale mechanistic information for biologically differential structure‐to‐function relationships.

## Experimental Section

### Preparation of Unilamellar Vesicles as Artificial Membranes

All the peptides were purified from chemical compounds such as trifluoracetic acid (TFA) and non‐reacted constituents, using high performance liquid chromatography. The templates and their characteristics are provided in Table 1.

Two types of artificial membranes were considered: zwitterionic unilamellar vesicles (ZUVs) and anionic unilamellar vesicles (AUVs), mimicking mammalian and microbial membranes, respectively. 1‐palmitoyl‐2‐oleoyl‐sn‐glycero‐3‐phosphocholine (POPC) and 1‐hexadecanoyl‐2‐(9Z‐octadecenoyl)‐sn‐glycero‐3‐phospho‐(1’‐rac‐glycerol) (POPG) were both from Avanti® Polar Lipids, Inc. Solvents (chloroform 99.8 %, Sigma; methanol >99.9 %, Sigma‐Aldrich) were of analytical grade. POPC was used to assemble ZUVs, whereas a mixture of POPC/POPG at 3 : 1 (molar ratio) (from here onwards abbreviated ‘POPC/POPG’), was used to assemble AUVs, based on a procedure published elsewhere^[67]^ (cf. SI, Section 1.1). The liposomes were suspended in 10 ml of 4‐morpholinepropanesulfonic acid buffer (pH∼7.4, MOPS, Sigma‐Aldrich). These lipid compositions yield fluid‐phase membranes at room and physiological temperatures. ZUVs’ and AUVs’ morphologies and chemical composition were analysed by photon correlation spectroscopy, scanning electron microscopy and UV/Vis spectroscopy yielding a mono‐modal size distribution of neutral (ZUVs) and negatively charged (AUVs) round‐shaped structures (cf. SI, Section 1.2).

### Preparation of Peptide Films, Lipid Films, and Lipid‐Peptide Films

For the preparation of peptide films, a peptide solution (c∼10 μM) was drop‐casted on a HDPE foil (d∼0.01 mm, Goodfellow). HDPE is a conventional substrate of high transparency used in the 600 to 10 cm^−1^ spectral range.[^37^, ^68^] We used this as a sample substrate as it has no spectral contributions that may overlap with those of peptides or lipid membranes in the considered FIR spectral window. We acknowledge however, the fact that it presents over the 400–0 cm^−1^ range, a very weak crystal lattice mode at 72 cm^−1^ (w). This does not constitute a disturbance in our studies, as the sample signal spectrum is systematically acquired against the (FIR source+foil) background and therefore automatically compensated. For the preparation of ZUVs and AUVs lipid‐bilayer films, the corresponding liposome solution was poured over an HDPE foil in a Petri dish and maintained under agitation for 2 hours to enable the deposition of the lipid bilayer onto the substrate surface.[^29^, ^30^] This deposition may lead to not strictly controlled bilayers patterns, with potential stacks of liposomes but still provide in average a good approximation of bilayers’ characteristics.

Working with thin films enables the study of interaction mechanisms between peptides and lipids, i. e. the incorporation of peptides into membranes. For the films of combined lipid‐peptide, the lipid films ZUVs and AUVs were first prepared as described previously. Afterwards they were incubated with 100 μl of peptide sample solution (c∼10 μM) (anionic QAELA, non‐AMP; and cationic KARLA, AMP, respectively) under controlled humidity (85 %) for 1 hour in a Petri dish to allow the peptides to bind and diffuse in the membranes according to published procedures.^[69]^ Humidity was maintained in a Tupper® box by a saturated potassium sulphate salt solution (K_2_SO_4_, 99.0 %, Merck) heated up to about 40 °C.^[70]^ Subsequently, the remaining non‐reacted and non‐adsorbed peptides were purged away methodically from the solutions by thorough washing steps with ultra‐pure water (15 MΩ⋅cm, Millipore GmbH). The peptide films, lipid films, and peptide‐membranes systems were all air‐dried prior their use for FTIR experiments. UV/Vis investigations on QAELA and KARLA peptide templates verified peaks originating from aromatic amino acids such as Tyrosine, Alanine and Leucine (cf. SI, Figure S2).

### FIR Studies on Peptides and Model Reconstituted Membranes

FIR investigations were carried on at the infrared beamline ‘IRMA’ of the PTB Metrology Light Source,[^71^, ^72^] operating at an electron energy of 630 MeV, with a maximum stored electron beam current of 190 mA. The irradiance of a synchrotron source in the FIR region can be as much as three orders of magnitude greater than conventional thermal sources (e. g. globar, Hg lamp, microwave sources), which leads to higher brilliance. High excitation radiation power, i. e. high photon flux, with high temporal and spatial stabilities is a prerequisite in the FIR spectral region to ensure low noise levels at the wavelengths of interest. The non‐invasive transmission geometry was preferred to conventional ATR reflectance measurements that are easily prone to spectral artefacts.

Transmission spectra were recorded between 400 and 40 cm^−1^ using a Vertex 80v FTIR‐spectrometer (Bruker Optics GmbH) fitted with a 6 μm Mylar beamsplitter, a liquid helium‐cooled Si bolometer detector configured with a 1000 cm^−1^ longpass filter cut, and an aperture setting of 12 mm. The peptide films, lipid films, and peptide‐membranes systems, air‐dried onto HDPE foil, respectively, were analysed by T‐dependent transmission experiments, conducted under steady vacuum (p∼1 mbar) in a helium cryostat (Oxford Instruments) in the temperature range 298–10 K to avoid any interference of water vapour.

As previously mentioned, the sample signal spectrum is systematically acquired against the background (FIR source, spectrometer setting, foil) in the same alignment conditions to avoid and automatically compensate potential artefacts (e. g. beamsplitter), which may distort the resulting sample spectrum. Each spectrum was collected with the Opus software V.7.2 (Bruker Optics GmbH) and consists of 512 averaged scans acquired with a spectral resolution of 4 cm^−1^. All interferogram scans were submitted to Blackman Harris 3‐Term window function and to a zero‐filling factor of 2 prior Fourier Transformation. Single channel spectra were normalised to the beam current using Origin 9.0G. The absorbance spectra discussed here were plotted without any baseline correction, smoothing or normalisation procedures to ensure a proper dataset for 2D correlation analysis. To increase the visibility of the T‐dependent spectral evolution, an arbitrary offset in absorbance (between 0.05 and 0.1 au) was applied between each spectrum of the presented dataset; the absorbance scale was kept valid only for the first spectrum (i. e. at T=298 K).

### 2D Correlation Analysis on Sample Film Spectra

2D correlation analysis provides a direct and quick visualisation of changing spectral domains upon specific external perturbations, as well as a reduction of the spectral complexity.[^52^, ^73^] It presents the advantage of being free of human inputs (e. g. deconvolution strategy, background correction, subtraction of the medium, etc.) and puts on display easily readable synchronous/asynchronous 2D correlation maps. 2D correlation analysis not only (i) enhances the spectral resolution and (ii) effectively resolves hidden information in overlapping peaks, but also (iii) allows for band assignments via correlation of bands selectively coupled by interaction mechanisms, (iv) unravels the reaction process by probing the specific order (asynchronous) or concomitance (synchronous) of spectral intensity changes, and finally (v) investigates various intra‐ and intermolecular interactions through selective correlation of the peaks, and therefore correlation of the reacting groups, including upon external factors (eg. temperature, concentration, etc.).^[52]^ It is therefore a powerful diagnostic tool which can extract and put on display much more information than classical spectral analysis. It was conducted on T‐dependent FIR spectral datasets in the 400–40 cm^−1^ spectral region and on the full T range (298 to 10 K) using the program 2Dshige v1.3 by Morita et al.[^73^, ^74^] For more details on the theory and interpretation, cf. to SI Section 2.3.

## Conflict of interest

The authors declare no conflict of interest.

1

## Supporting information

As a service to our authors and readers, this journal provides supporting information supplied by the authors. Such materials are peer reviewed and may be re‐organized for online delivery, but are not copy‐edited or typeset. Technical support issues arising from supporting information (other than missing files) should be addressed to the authors.

Supporting Information

## Data Availability

The data that support the findings of this study are available in the supplementary material of this article.
