# Supplementary material for: Investigating Membrane‐Mediated Antimicrobial Peptide Interactions with Synchrotron Radiation Far‐Infrared Spectroscopy
Source: Chemphyschem. 2022 Jan 14;23(4):e202100815. doi: 10.1002/cphc.202100815 (PMC9303692; doi:10.1002/cphc.202100815)
Supplement: Supplementary file 1 — Supporting Information [file CPHC-23-0-s001.pdf]

ChemPhysChem

Supporting Information

Investigating Membrane-Mediated Antimicrobial Peptide Interactions with Synchrotron Radiation Far-Infrared Spectroscopy

Andrea Hornemann,* Diane M. Eichert,* Arne Hoehl, Brigitte Tiersch, Gerhard Ulm, Maxim G. Ryadnov, and Burkhard Beckhoff

Supporting Information (SI)

Contents

1. Experimental Section 1
1.1 Preparation of unilamellar vesicles as artificial membranes 1
1.2 Cryo-SEM analysis, Dynamic Light Scattering, and UV/Vis spectrophotometry 2
1.3 Preparation of sample pellets for FTIR experiments 3
1.4 MIR study of antimicrobial peptides in solution 4
***2.* Results and discussion** 5
2.1 Cryo-SEM analysis, DLS and UV/VIS Spectroscopy 5
2.2 FIR signatures of HDPE-embedded peptide pellets 7
2.3 Theory of 2D correlation spectroscopy 8
2.4 Studies on the reversibility process of T-dependent FIR on peptide 9
2.5 FIR specifics of KARLA (AMP) and QAELA (non-AMP) peptides 10
2.6 ZUVs and AUVs - Model membranes' specificities 10
2.6.1 Model membranes' specificities 10
2.6.2 Asynchronous results of 2D correlation analysis on ZUV and AUV membranes 11
2.7 FIR analysis of the ZUVs put in presence of QAELA and KARLA peptides 12
2.7.1 FIR Spectra 12
2.7.2 2D correlation analysis of QAELA and KARLA with ZUV membranes 13
2.8 MIR studies on antimicrobial peptides in solution 19
3. References 22

1. Experimental Section

1.1 Preparation of unilamellar vesicles as artificial membranes

Two types of artificial membranes i.e. zwitterionic unilamellar vesicles (ZUVs) and anionic unilamellar vesicles (AUVs), mimicking mammalian and microbial membranes, respectively, were prepared from two liposome components: 1-palmitoyl-2-oleoyl-sn-glycero-3-phosphocholine (POPC) and 1-hexadecanoyl-2-(9Z-octadecenoyl)-sn-glycero-3-phospho-(1'-rac-glycerol) (POPG). POPC was used to assemble ZUVs, while a POPC/POPG 3:1 molar ratio was used to assemble AUVs. Both liposomes were from Avanti Polar Lipids, Inc; the

solvents (chloroform 99.8%, Sigma; methanol >99.9%, Sigma-Aldrich) were of analytical grade.

In short, 52.2 mg of POPC or 35.5 mg / 12 mg POPC/POPG (3:1 molar ratio), from here after referred to as POPC/POPG, were weighed in a round-bottom flask, respectively, then incorporated to a chloroform/methanol solution (2:1, v/v). The corresponding flask was attached to a rotary evaporator (Rotavac Valve®, Heidolph Instruments) and rotated to about 60 rpm in a water bath (T~50 °C) until complete dissolution of the lipid. Then, the vacuum level was set at ~100 mbar, and rotatory evaporation was pursued until eradication of the liquid from the solution, leading to a dry lipid thin film on the walls of the flask which was maintained under vacuum for an additional 2 hours. Consequently, 5 ml of 3-(N-morpholino) propanesulfonic acid buffer solution (pH~7.4, 0.15 M NaCl, MOPS buffer grade, Sigma Aldrich), and 0.5 g of glass beads (Ø~2 mm) were added to the flask before a new evaporation cycle overnight (ca. 60 rpm, room temperature and constant pressure ~100 mbar). This ensured that all lipids were detached from the flask wall, and are present in the resulting homogeneous milky white suspension.

The hand-held extrusion process was carried on a hot plate at about 60 °C by loading one of the two syringes with ca. 500 µl of lipid vesicle solution and passing it through the syringes about 21 times (an odd number of passes is necessary for finishing the extrusion on the opposite syringe), filtering thoroughly and consecutively in the first syringe from the same side of the filter. The size of liposomes was thus adjusted to maximum 50 nm with a 50 nm pore size Whatman® filter. After extrusion, liposomes were kept in a brown flask and stored in the fridge until their experimental use.

1.2 Cryo-SEM analysis, Dynamic Light Scattering, and UV/Vis spectrophotometry

In brief, DLS, cryo-SEM, UV/Vis spectroscopy ascertained the correct size distribution and spherical shape of the liposomes, the hydrodynamic diameter of which was in the 190 nm to 210 nm range. In addition, ζ potential measurements confirmed the expected surface potentials (neutral for ZUVs and negative for AUVs). Further UV/Vis investigation on the QAELA (non-AMP) and KARLA (AMP) peptides validated the correct amino-acid composition of the peptides.

For Cryo-SEM and TEM analysis, a small amount of the liposome suspension, either ZUVs or AUVs, was placed in a copper sandwich holder and successively plunge-freezed into a nitrogen slush at atmospheric pressure. Freeze-fracturing was carried out in a cryo-preparation chamber (Gatan Inc. Alto 2500) at -180 °C. After fracturing, the temperature of the samples was increased to -98 °C for the freeze-etching procedure (45 s) and then re-decreased to -120 °C for sputtering them with platinum (Pt). Afterwards, the samples were transferred into the Cryo-Field Emission-SEM S-4800 (Hitachi) and SEM micrographs were acquired at a stage temperature of -145 °C and an accelerating voltage of 2 kV.

For the preparation of replicas, a small amount of the liposome suspension was placed in a Cu-Cu-specimen sandwich holder and plunge-freeze into liquid propane cooled by liquid nitrogen at atmospheric pressure. Freeze-fracturing was carried out in a freeze fracture apparatus BAF 400 (Balzers, Liechtenstein) at -150 °C. After fracturing, the temperature of the sample was increased to -100 °C for the freeze-etching (60 s) before Pt/C evaporation. The replicas were cleaned in sulphuric acid and washed in distilled water, mounted on uncoated copper grids and examined in a transmission electron microscope JEM-1011 (JEOL) at an accelerating voltage of 80 kV.

Particle diameters of both ZUVs and AUVs suspensions were studied by Dynamic Light Scattering (Zetasizer Nano, Malvern Instruments). The measurements were carried out in quartz microcuvettes (Brandt®, HELLMA) by averaging 10 measurements of 15 runs according to a cumulative fit procedure.

For UV/Vis analysis on KARLA and QAELA, a solution of 1% was prepared using phosphate buffered saline (PBS). The sample solutions were dropcasted onto HDPE foil (d=0.01 mm) and analysed in the 200-550 nm spectral window. The absorption spectroscopy of these liposome suspensions was performed in quartz cuvettes (Brandt®, HELLMA) with a UV/Vis-NIR spectrophotometer system (Jasco) in the 190-800 nm spectral range.

1.3 Preparation of sample pellets for FTIR experiments

Sample pellets of approx. d~10 mm (thickness ca. 0.3 mm) were prepared for infrared analysis mixing the peptides with high density polyethylene powder (HDPE, d~53-75 µm, Sigma-Aldrich). The amount of sample required depends on the sample itself and on its relative absorption in the infrared spectral range. 30 mg of sample with 30-50 mg of HDPE powder was found adequate for preparing a pellet which complies with the infrared analysis requirements in the FIR spectral range. The HDPE powder was ground with a ball mill (RETSCH) to reduce the HDPE particles to a size of about 1-2 µm. A hydraulic press

(MSScientific Chromatographie Handel GmbH) was tuned to a pressure of about 7 tons and pressing time of 10 min for all HDPE and HDPE-sample pellets preparations.

1.4 MIR study of antimicrobial peptides in solution

For the control experiments on the conformation state (e.g. helicity) of AMPs, mid-infrared (MIR) spectral investigations were conducted in liquid state and in transmission geometry. The peptide sample solution (c~1% in phosphate-buffered saline PBS) was sandwiched in between two calcium fluoride windows; in one was etched a channel of 6 μm depth to define the peptide layer thickness, the other one served as coverslip. Background scans were collected before sample measurement from PBS buffer solution and rationed against the sample spectrum.

Data acquisition parameters for the MIR investigations were the same then the ones used in FIR spectral investigations (cf. to the Experimental Section in the main manuscript).

2. Results and discussion

2.1 Cryo-SEM analysis, DLS and UV/VIS Spectroscopy

Cryo-SEM micrographs (Figure S1, insets) reveal that the liposomes' vesicles present a round morphological shape and a diameter of about 200 nm. This result is in accordance with the UV/Vis extinction spectra (Figure S1, top) obtained, which show that the hydrodynamic diameters of ZUVs and AUVs liposomes (c~1.56% in MOPS) are quite similar, residing between 190 to 210 nm, and which display an absorbance maximum at about λ_{max} (ε_{ZUV}, ε_{VUV})=193 nm (487 $mol^{-1}\,dm^{3}\,cm^{-1}$, 346 $mol^{-1}\,dm^{3}\,cm^{-1}$), in line with the chemical composition of the phospholipids. Although the DLS spectra (Dynamic Light Scattering, Figure S1, bottom) also indicate a mono-modal size distribution, the hydrodynamic diameters for ZUVs and AUVs liposomes are significantly different: 242 and 147 nm, respectively. These discrepancies, in comparison to the UV/Vis values, are due to the sensitivity of the DLS method to the overall size distribution of various liposomal structures, including aggregates, that cause a higher light scattering than smaller un-aggregated or more regular liposomes.

The ζ potential measurements are consistent with the theoretical assumptions of the considered liposomal surface charges, showing that the surface charge of the neutral ZUVs liposome formulation is about -1.46 mV, whereas the ζ potential of the negatively charged AUVs liposome is about -46.8 mV.

UV/Vis investigations (Figure S2) on QAELA (non-AMP) and KARLA (AMP) peptide templates reveal a massif of peaks of an absorbance maximum located around λ_{max} (ε_{KARLA}, ε_{QAELA})=275 nm (692 $mol^{-1}\,dm^{3}\,cm^{-1}$, 571 $mol^{-1}\,dm^{3}\,cm^{-1}$). These peaks derive from aromatic amino acids such as Tyrosine,[1] Alanine and Leucine[2] and can be observed in all absorbance spectra of these peptides.

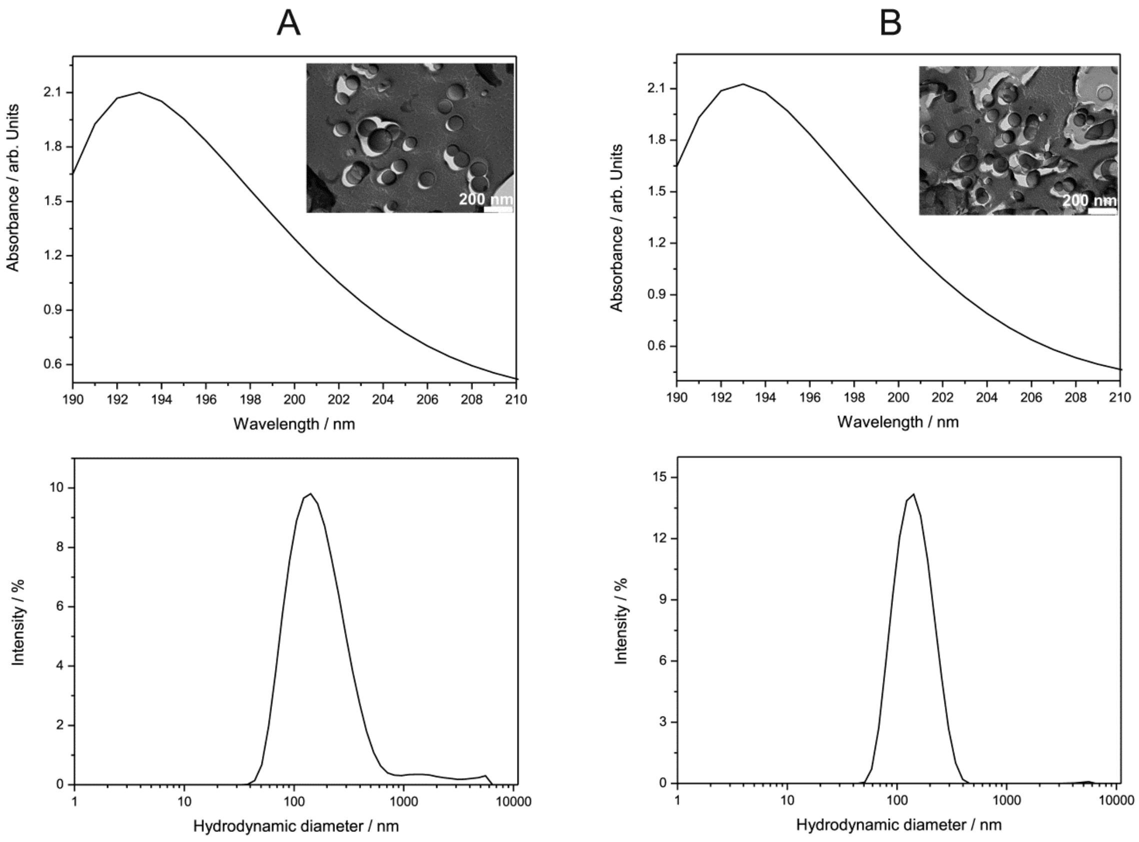

Figure S1. top: UV/Vis extinction spectrum (A) ZUVs and (B) AUVs, and **bottom:** corresponding DLS spectra. The insets show cryo-SEM images of (A) replicas and (B) of cryo-fractions (B) for the respective liposomes.

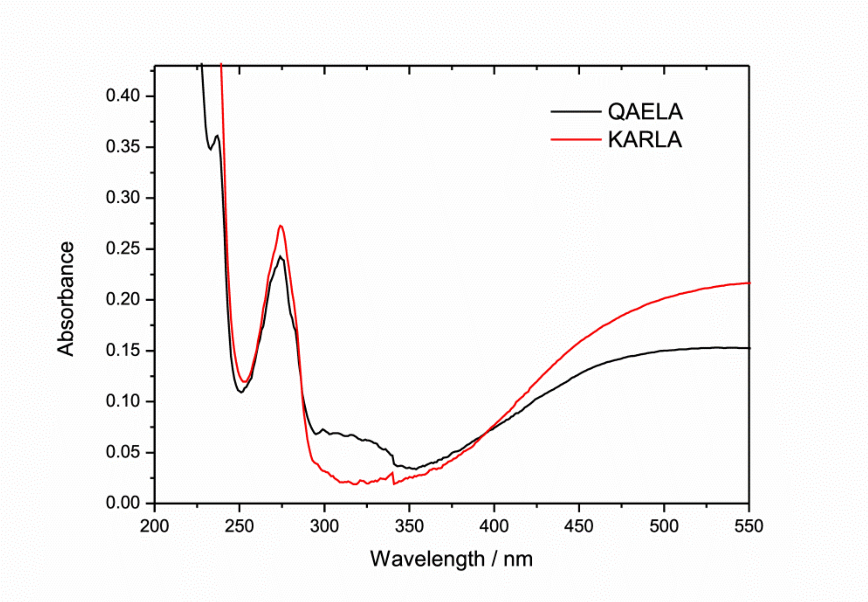

Figure S2. UV/Vis spectrum of peptide films (1% used) prepared on a HDPE foil.

2.2 FIR signatures of HDPE-embedded peptide pellets

The pellets spectra entail broad spectral features in contrast to the sample film signatures (Figure S3). This fact demonstrates the importance of sample preparation. Indeed, dilution in an external medium and grinding may modify the charge of the peptide, force the peptide to acquire a different orientation due to the medium, or change conformation. All these artefacts may alter significantly the integrity of H bonds, broaden the signal, and therefore compromise the reliability of the measurements.

There are little observable T-dependencies of the non-AMP (QAELA) peptide. Some non-regular T-dependencies can however be noted for the AMP (KARLA) peptide, with some changes in the global shape and bands position. These observations denote of a limited perturbation of the compounds upon cooling, and therefore a restricted re-organisation and movement potential of the various intra- and intermolecular interactions. However, some information about the conformation of the peptides, and in particular the band related to the collective backbone mode region (Amide VII) are still available. The main broad band at ca. 145 cm^{-1} (br) is typical of α-helical conformation and the one at ca. 100 cm^{-1} (w, vw) of random coils conformation and intrinsic bonds of hydrogen bonds. The band at ca. 265 cm^{-1} (m) is also linked to the peptide backbone modes.

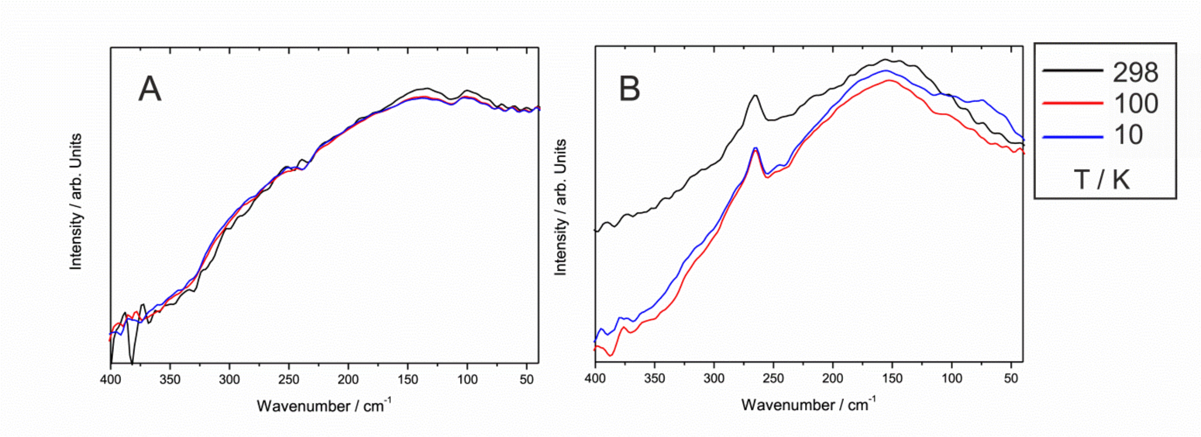

Figure S3. FIR signatures of QAELA (non-AMP) peptide (A) and KARLA (AMP) peptide (B) sample pellets with HDPE as embedding medium. Studies were performed at 298, 100 and 10 K, respectively. Experiments were performed in the spectral range between 400 and 40 cm^{-1}.

2.3 Theory of 2D correlation spectroscopy

2D correlation is based on the calculation of the covariance which is a measure of how much two random variables change together.[3] The 2D correlation analysis enables a profound study on the relationship of infrared spectral datasets that can indicate an increasing/decreasing peak intensity due to an external perturbation such as (i) T-induced changes in molecular structure or (ii) modifications on components. For instance, 2D correlation has been exploited for resolving T-dependent spectra of helix-forming peptides[4] and probing protein unfolding processes.[5]

The 2D correlation on T-dependent IR data provides two complementary signals, the synchronous and asynchronous spectrum, hence enabling to determine in-phase events that occur at the same time and out-of-phase events that occur at different times, respectively. It allows to determine the sequence of spectral changes, to identify various inter- and intramolecular interactions and band assignments of reacting groups, as well as to detect correlations between spectra of different techniques such as Raman spectroscopy. A particular 2D correlation pattern can derive from spectral events such as band shifts and/or overlaps of which the intensity changes in the opposite direction, as well as band broadening. The direction of intensity change is represented by calculation of synchronous spectra, whereas the asynchronous spectrum determines the sequence of intensity changes that can be probed.[6] Further theoretical explanations for calculation of the synchronous and asynchronous correlation amplitudes can be found in refs. [7,8]

Following rules apply to synchronous/asynchronous 2D correlation maps:

(i) The synchronous 2D cross-peak at the coordinate (x,y) is positive (highlighted in red Φ) if the intensities of the bands at x and y in the dataset are changing in the same direction. (ii) If the intensities of the bands at x and y in the dataset are changing in the opposite direction, the synchronous 2D cross-peak at the coordinate (x,y) is negative (highlighted in blue Φ). The 2D synchronous spectrum is symmetric relative to the main diagonal that contains positive peaks, also called auto-peaks. The main diagonal signal is referred to as autocorrelation signal. The off-diagonal cross-peaks can be either positive or negative.

On the other hand, the asynchronous spectrum is asymmetric and never has peaks on the main diagonal. Providing that the sign at the corresponding position in the synchronous plot is positive (iii) if the change at x mainly precedes the change in the mode at y, the asynchronous 2D cross-peak at (x,y) is positive, and (iv) if the change at x mainly follows the change in the band at y, the asynchronous cross-peak is negative. If the sign at the corresponding position in the synchronous plot is negative, points (iii) and (iv) are reversed.

2.4 Studies on the reversibility process of T-dependent FIR on peptide

The T-dependent FIR series reported here for the QAELA peptide (non-AMP) reveal a perfect reversibility of the molecular structure after a cycle of cooling or heating, ascertaining the non-dependency of the temperature treatment onto the results (Figure S4).

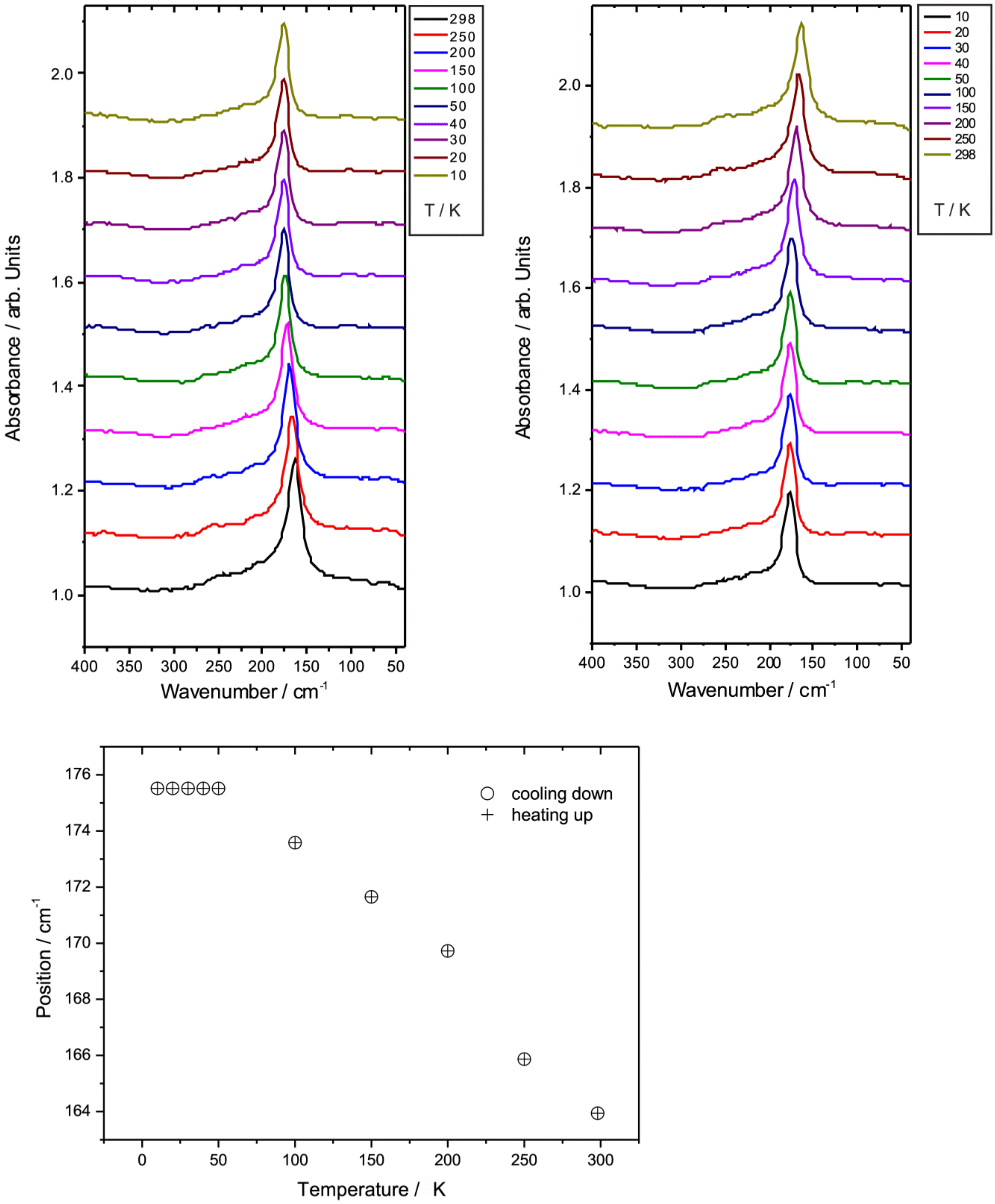

Figure S4. Reversibility studies of QAELA (non-AMP) peptide sample film spectra measured with a cryostat in the 298-10 K temperature range upon cooling (circles °), and upon heating from 10 to 298 K (crosses +). Studies were performed in the 400-40 cm^{-1} spectral range.

2.5 FIR specifics of KARLA (AMP) and QAELA (non-AMP) peptides

Around 75 cm^{-1} (w) one can observe bending modes of H_2O, readily visible in the anionic QAELA peptide spectrum (see Figure 2A).[9] Both QAELA and KARLA peptides display a strong mode at about 170 to 175 cm^{-1} (s) which can be assigned to the torsion modes (-CO_2H) of alanine residues in the peptide,[10] together with a torsion mode that derives from the C–N bond. They also show a mode by about 220 cm^{-1} (w), a result of a torsion bands and amino acid lattice rotational modes.[11,12] Another mode at about 240 cm^{-1} (w) can be identified among others as a methyl-specific torsion mode.[13,14]

The QAELA and KARLA spectra are accompanied by a series of modes between 265 (w) and 215 cm^{-1} (vw), which most probably originate from the other AMP's amino-acids (e.g. glutamic acid and lysine), and whose exact contributions would require dedicated studies and simulations.

2.6 ZUVs and AUVs - Model membranes' specificities

2.6.1 Model membranes' specificities

Upon cooling, the FIR signatures of neutral (mostly) ZUV membranes (Figure 3A) show changes in intensity in the spectral regions at ca. 175 to 165 cm^{-1} (s), with a noticeable shift of the mode at ca. 165 cm^{-1} (s) to higher frequency, i.e. to ca. 177 cm^{-1} (s), and at 210 to 200 cm^{-1} (w/m). These changes can be attributed to intermolecular hydrogen bonding structure and to the torsional mode of the hydrocarbon chain from one side, and to other torsional modes of the hydrocarbon chain from the other side, respectively. Another shoulder related to a torsional mode of the hydrocarbon chain (τ–CH_2) arises at ca. 220-210 cm^{-1} (m) for temperatures lower than 200 K, and is most pronounced for T=10 K. This indicates some important modifications of the liposome backbone with T. Despite the above-mentioned similarities, the FIR signatures of anionic AUV membranes (Figure 3B) clearly differ from those of ZUV membranes. For instance, in the spectral region between 250 and 150 cm^{-1}, as well as in between 375 and 325 cm^{-1}, where a mode referring to torsions of the C–N of choline groups occurs. A behaviour similar to the one found for ZUV membranes occurs for AUV membranes T-dependent bands at ca.178-166 cm^{-1} (s) and at about 208 cm^{-1} (m). The main bands at ca. 178-166 cm^{-1} (s) develop an additional defined shoulder at ca. 233 cm^{-1} (w). A broad mean band related to hydrocarbon chain backbone at about 183-170 cm^{-1} (m) is also detected. The shifts observed in between the bands' positions of both types of membranes related to their main (and mostly identical) molecular groups reflect significant differences in the molecular structure. These shifts may be remotely linked to the overall charge positioning offered by these molecules

within or at the surface of the liposome film layers, or differences in solvation of the polar or head groups, i.e. linked to their binding affinities.

Table S1. Far-infrared modes of the investigated ZUV and AUV membranes.

Mode / cm^{-1}	Tentative assignments	Refs.
40–58 (br,w)	intra- and intermolecular H bonds, ν–OH, H intermolecular librational and bending modes	[9,16]
83 (br,s)	bending mode of H_2O	[9,15–17]
94–97 (w)	intrinsic mode of H bond, hydrogen bonds	[18–20]
110–115 (w)	intermolecular bending and librational modes, C–CH_3 motions, N–H…O	[12,17,21,22]
132 (m)	intrinsic mode of H bond	[15,18,20]
164-176 (s)	τ–C–CH_2 hydrocarbon chain	[18,20]
183-191 (s,m)	τ–C–CH_2 hydrocarbon chain	[15,18,20,22]
210–220 (m)	H bond stretching, τ C–CH_2 chain	[12,18,20,22]
231 (m)	τ–CO–NH, τ –C–CH_3	[12,20]
241 (w)	τ–C–CH_3	[18,20]
250–275 (w)	τ–C–CH_3, τ-C-C-N, δ–C–C–N, δ–C–N–C	[20,22]
341 (m)	τ–C–C–N, hydrocarbon chains and skeleton vibrations	[18,20]
355 (m)	τ–C–C–N, hydrocarbon chains and skeleton vibrations	[18,20]
368–380 (m,w)	τ–C–N of choline group, τ–C–C–N, hydrocarbon chains and skeleton vibrations	[18,20]

2.6.2 Asynchronous results of 2D correlation analysis on ZUV and AUV membranes

The spectral features put in evidence in the synchronous maps (Figure 3C, Figure S3D) are equally resolved in the asynchronous spectrum in the case of neutral ZUV membranes (Fig. S5A), and slightly better resolved in the case of anionic AUV membranes (Figure S5B). Cross peaks are developed at wavelengths corresponding to individual bands related to the discussed molecular groups. For both membranes, it is again the bands related to the inter-

layer hydrogen water bridges arising from head (phosphocholine) or polar groups, together with torsional modes of the backbone (to which are attached the phosphocholine head groups) at 175 (s) and 165 cm^{-1} (s) which are sharing cross-peaks mostly with the backbone chain torsional groups, and with the various types of H-bonds, which contribute much more in the case of AUV membranes. These asynchronous results put in evidence that the changes occurring for these specific torsional deformation of the backbone (τ–C–CH_2) precedes the changes taking place for other torsional modes of the hydrocarbon chains and for the intra-, intermolecular and intrinsic H bonds, these two later kinds being mostly concomitant. For AUV membranes another level of dependency can be outlined, as torsional deformations of the carbon chain (at ca. 165 to 200 cm^{-1}, CH_2) slightly precede other torsional deformations of the backbone chain (C-C-N). Altogether, these results help in deconvoluting the numerous overlapped contributions, highlight the partial co-dependency in the molecular groups evolutions, and determine the sequence of T-changes happening to these spectral moieties.

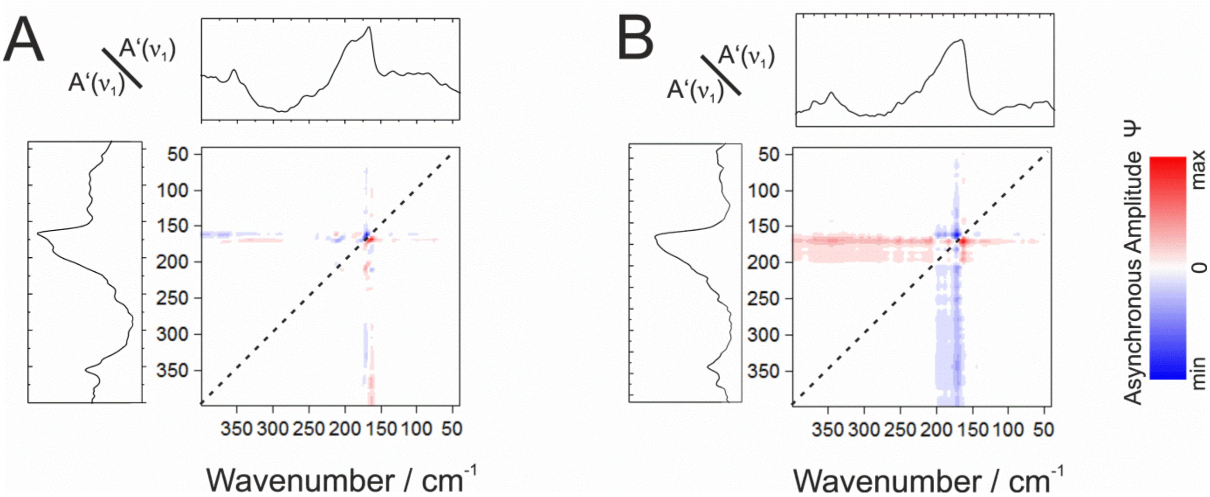

Figure S5. 2D asynchronous maps of (A) ZUV and (B) AUV membranes. in the 298-10 K T-range. Spectra in A and B are displayed exemplarily for T=298 K, and are not the average of all spectra contained in the studied range.

2.7 FIR analysis of the ZUVs put in presence of QAELA and KARLA peptides

2.7.1 FIR Spectra

The T-dependent FIR signatures for the ZUVs mammalian model membrane exposed to the QAELA (non-AMP) and KARLA (AMP) peptides are displayed in Figures S6A and S6B, respectively. Very limited variations can be observed and no defined peptide-membrane

interaction band can be resolved, the signal remaining mostly at the noise level. No re-organisation of water molecules or other hydrogen bonds of ZUV membranes or of QAELA was assignable.

This demonstrates that QAELA remains unfolded and repulsed in presence of ZUV membranes which is mostly neutral. Indeed, CD spectra (data not shown here) for the anionic QAELA peptide comprise an ensemble of disordered conformers in solution (minimum at ~200 nm). However, for the KARLA-ZUVs (AMP–ZUVs) system, a certain level of interaction could be probed as we could assign a slight FIR signal increase between 250 and 170 cm^{-1} (compare Fig. S6A and S6B) which includes T-evolving (H-bonds) contributions both from the peptide and the torsional modes from hydrocarbon chains and τ-C-C-N groups coming from the ZUV membranes (Table 1 and Table S2). Nevertheless, this processus is far from being favourable due to charge domains, and its occurance remains rather limited, as KARLA remains predominantly unfolded. CD measurements also confirmed a slight interaction between KARLA and ZUV membranes being probed by a band at ~214 nm, indicating some alpha-helical conformation.

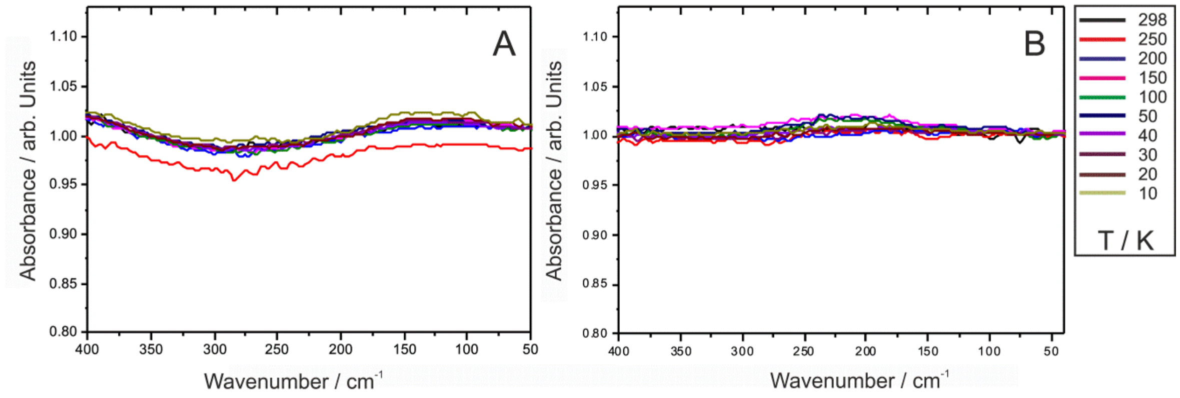

Figure S6. FIR signatures of (A) ZUV mammalian model membranes with QAELA (control non-AMP, anionic) peptide and (B) ZUV membranes with KARLA (AMP, cationic) peptide. Temperature range: 298-10 K. Experiments were performed in the 400-40 cm^{-1} spectral range. The absorbance scale (A.U.) was kept identical for all the peptide/membrane systems for ease of comparison between the spectra.

2.7.2 2D correlation analysis of QAELA and KARLA with ZUV membranes

2D correlation analysis were realised for consistency in between the data sets as well as an attempt to evidence some potential patterns in the low noisy signal obtained. However, cross-

correlating 2 signals at (or almost at) the noise level leads to some artefacts such as auto-peak or cross-peak phantoms, as well as blur of part or of all the spectral regions, and impose some thorough and critical check of the correlation results. This was observed in both the synchronous and asynchronous plots, as well as for both 2D auto- and cross correlation.
In the case of the 2D auto-correlation of the KARLA (AMP) and QAELA (non-AMP) peptides with neutral ZUV membranes (also valid for the system QAELA-AUVs), the analysis is unreliable due to the little variations emerging from the noise of the resulting spectra and those are therefore not displayed.

2.7.2.1 2D synchronous / asynchronous cross-correlation analysis

Despite the above mentioned limitations for 2D correlation analysis due to the spectral noise, 2D cross-correlation analysis between the signal of the peptide versus the signal of the peptide-membrane system is nonetheless useful as it highlights the molecular variations with T of the peptide, and of its Amide VII band in particular, in the considered peptide-membrane system. For the 2D correlation maps reported in Figures S7A and S7B, only streak elongated peak are visible (as expected) at the Amide VII coordinates of the related peptide (mostly).

2.7.2.2 Control peptide (QAELA) with mammalian model membranes (ZUVs)

For QAELA (non-AMP) versus ZUV membranes and QAELA, 2 main auto-peaks at ca. 180 and 165 cm^{-1}, and a faint auto-peak at ca. 211 cm^{-1} are noticeable in the synchronous plot (Figure S7A), as well as a diffuse (red) trail from about 235 to 175 cm^{-1}. The elongated streak lines visible at the wavelengths characteristic of the backbone band (and Amide VII) with associated H bonds are evolving simultaneously in opposite direction (red at ca. 180 cm^{-1}, and blue at ca. 165 cm^{-1}), and are correlated synchronously with the entire spectral range probed, i.e. also with the other molecular groups forming the peptides, such as the hydrocarbon chains.

The asynchronous plot (Figure 7C) indicates no order in the sequence of events, apart from the modification at ca. 170 cm^{-1} which may precede all others. This analysis remains, consequently, inconclusive. No integration of the QAELA peptide in the ZUV lipid membranes can be put in evidence as no increase of the Amide VII mode contributions or decrease of the ZUVs' torsional modes of the hydrocarbon chain, respectively, occurs. In addition, no re-organisation of the water molecules of ZUV membranes or of QAELA (domains below 150 cm^{-1} of molecular breathing), or generally speaking of the H bonds i.e., no modification of the liposomes leaflets, is detected.

2.7.2.3 Antimicrobial peptide (AMP) with mammalian model membrane (ZUVs)

For the synchronous 2D cross-correlation map of KARLA (AMP) versus KARLA and ZUV membranes (Figure S7B), the results are somehow opposite, as some diminution of the absorption characteristics for the torsional hydrocarbon chains of the liposome backbones are most likely represented by the blue auto-peak at ca. 180 cm^{-1}, whereas the red auto-peak at ca. 165 cm^{-1}, with a trail expending to ca. 150 cm^{-1}, and a diffuse streak elongated lines from ca. 135 to 60 cm^{-1}, could be explained by a small change of conformation of the the Amide VII (which presents an additional characteristic band at ca. 132 cm^{-1}), which is also correlated to changes of the H bonds and thus, of a potential but small level of insertion of the KARLA peptide inside the membrane. These modifications are albeit extremely limited considering the low FIR signal obtained with the AMP-ZUVs system (Figure S6B). The complex domains below 150 cm^{-1} are also significantly affected and refer, as already mentioned, to the various water domains and various H bonds present in the system, which proves a certain re-organisation of the system AMP-ZUVs. If one compares Figure 5B to Figure S7B however, a quite similar 2D correlation patterns can be observed. These may reflect a slight change in the conformation of the KARLA peptide as a structural adaptability response to the presence of the ZUV membranes.

The asynchronous plots of KARLA (AMP) and ZUV membranes with KARLA as control, (Fig. S7D) only reveal that the modifications around the Amide VII bands are correlated with the entire spectral domain probed. These do not put in evidence any precedence in the changes occurring to the molecular groups, these bands being “following” any modification that is taking place, and in this regard this 2D correlation analysis does not allow to draw any conclusion. If one compares Figure 5D to Figure S7D however, the similarity in the 2D correlation patterns is striking, once again. This observation is also consistent with the patterns observed in the synchronous plots and these may, again, reflect a change in the conformation of the KARLA peptide as a structural adaptability response to the presence of the ZUV membranes.

2.7.2.4 Summary

To sum up, no interaction of the QAELA (non-AMP) and no or limited interaction of the KARLA peptide with the mammalian model membranes (ZUVs) can be observed, as there is no and/or very tiny indication of detectable peptide-specific modes, respectively. Hence the negative

control template QAELA does not carry out any functionalities or interactions with the ZUV membranes, or little interactions in the case of KARLA (AMP). This can be partially explained by their physicochemical properties which entail an anionic and cationic characteristic, respectively. The QAELA and KARLA peptides possess their own main charge (negative and positive, respectively) but also domains of opposite charge which might help (for KARLA) or further limit (for QAELA) the incorporation of the peptides in the leaflets of the membranes due to favourable trans-potential. Although limited and weak, as it is mostly due to hydrophobic interactions (orders of magnitude lower and inefficient compared with electrostatic interaction), this level of attraction in between the AMP KARLA and the mammalian model membranes (ZUVs) may become significant and high enough, despite low binding affinities, to induce incorporation and perturbation of the lipid bilayer, including within neutral membranes as ZUVs. The degree of hydration of the interlayer –hydrogen water structures could also play a role, as demonstrated,[23] and facilitate transport of molecules inside phospholipidic membranes, even neutral ones. In *in vivo* systems however, the transmembrane potential can be more negative for the bacterial membrane than for the mammalian membrane, the first one will be hereby more prone to AMP's attack.

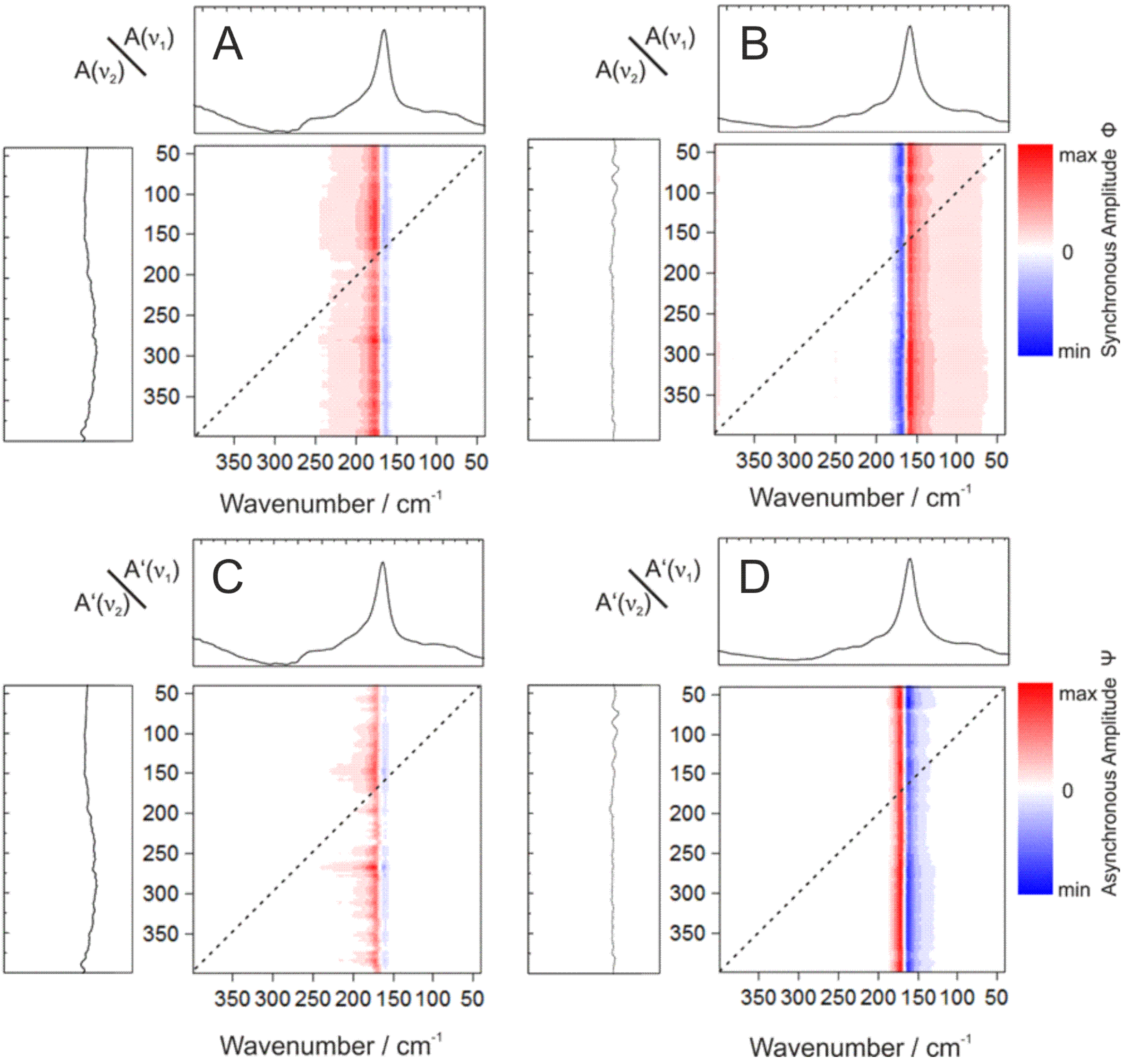

Figure S7. Synchronous and asynchronous 2D cross-correlation maps of (A, C) A(v_1) QAELA versus A(v_2) ZUV membranes with QAELA and (B,D) A(v_1) KARLA versus A(v_2) ZUV membranes with KARLA. Spectra are displayed exemplarily at T=298 K, and are not the average of all spectra collected in the studied range.

2.7.3 2D cross-correlation studies of QAELA (non-AMP) with AUVs lipid membranes

Similarly to the systems QAELA-ZUVs and KARLA-ZUVs, no 2D auto-correlation is presented for the QAELA-AUVs system as the signal is embedded in the noise. The QAELA-AUVs system 2D cross-correlation patterns reveals, as shown in Figure S8A by the synchronous plot (left), that the Amide VII mode region is evolving almost alone, with strong auto-peaks at 165 (blue) and 175 cm^{-1} (red), developing in opposite direction, and a less intense at ca. 215

cm^{-1} (red), upon which an elongated interaction domain extending to 240 cm^{-1} is visible. The Amide VII domain associated with H bonds is evidently varying with the other molecular groups which constitute the peptide, e.g. variation of skeletal deformation of the Amide VII mode, as expected during a change of conformation. No characteristic cross-peaks, as for QAELA with ZUV membranes (Figure S7A), can be observed for QAELA vs. QAELA with AUV membranes which would be assignable to H_2O bending modes and related H-bonds re-organisation, evidencing no interaction between AUV membranes with the negative control QAELA peptide. The asynchronous plot (Figure S8B) does not allow, as it was already the case for the QAELA-ZUVs system (Figure S7A), to define any order in the sequence of molecular changes with T. It only confirms that the T-changes are affecting the peptide itself, as it is obvious for the QAELA collective group modes, but it does not indicate any interaction with the ZUV membranes.

All considered, the features composing these synchronous and asynchronous plots appear to be very similar, with respect to their positions and patterns, to those being observed for the QAELA-ZUVs system (Figure S7A, Figure S7C). This is another indication that this negative peptide control template does not carry out any functionality or interaction with this type of lipid bilayers due to its physicochemical properties, for instance its anionic characteristics, which might induce a net charge repulsion. In addition, the presence of glycine residue is known for being a “helix breaker” in water solution, and to provide high flexibility to the molecule. QAELA was indeed found to be relatively insoluble in water, which further suggests the non-accessibility and electrostatic repulsion of its binding sites to water and/or external medium. Although it requires further investigation, FIR might be one of the techniques which helps to monitor the affinity of a given peptide with a given membrane in solution. In such, in presence of AUV membranes, and as it does in presence of ZUV membranes, the QAELA peptide may displayed some conformational flexibility to stabilise itself, without interacting effectively with the membrane.

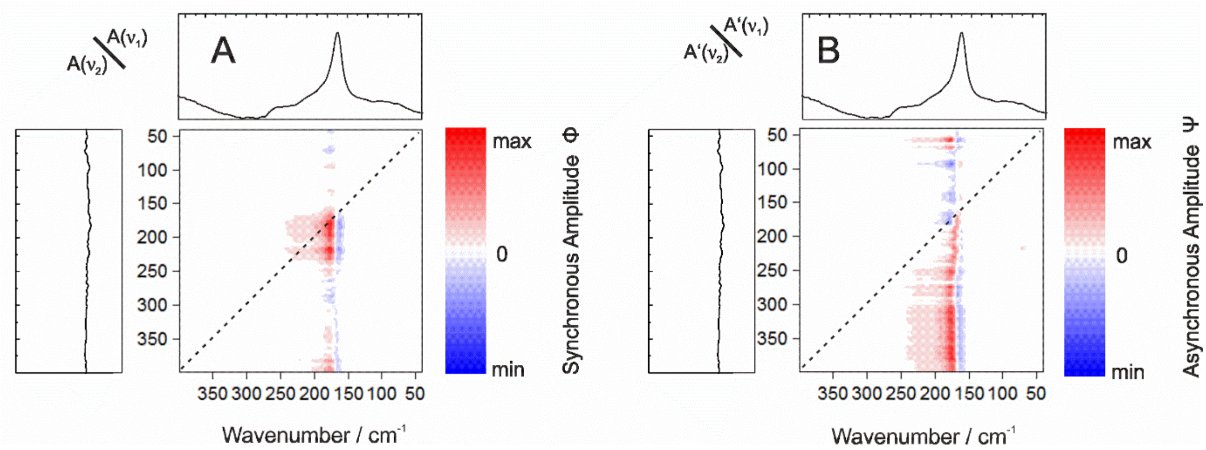

Figure S8. Synchronous (A) and asynchronous (B) 2D cross-correlation maps of AUV membranes with QAELA (non-AMP). Spectra are displayed exemplarily at T=298 K and are not the average of all spectra collected in the studied range.

2.8 MIR studies on antimicrobial peptides in solution

MIR studies were performed in order to probe the backbone structure of peptides that is linked to the peptide conformation state. The results of the measurement which were realised in liquid state are consistent with those obtained with the cryostat in the FIR spectral region, although investigations on sample thin films were conducted in this case. We infer these observations from the high similarity between both measurement configurations and experimental conditions, as both experiments took place under transmission geometry in complementary spectral ranges, namely FIR and MIR. The peptide solution (1%) was sandwiched between the two calcium fluoride windows that served as two MIR-transparent interface boundaries, and enabled probing the whole sample volume, whereas the peptide sample thin film was investigated on one FIR-transparent HDPE substrate.

The potential conformational level reached by the peptides while orienting themselves with respect to the surface(s) of the support, i.e. on CaF_2 windows for MIR and as thin film on HDPE foil for FIR, can be considered in first approximation the same, which enable a direct comparison in between the data resulting from the two different spectral domains and is an insurance of the relevance of the outcomes.

Figure S9 shows the Amide region in the mid-infrared spectrum of KARLA peptide, which is principally composed by a broad, featureless Amide I band centred nearby 1650 cm^{-1} (s), characteristic of an unordered structure, and thus of a primarily unfolded peptide. The Amide

I band position, together with substructure elements were evaluated[24–26] by the second derivative spectrum. The band at 1649 cm^{-1} (s) is indicative of mainly an unfolded conformation together with α-helical contributions, which lower-to-normal frequency tends to indicate a solvent exposed helix. This is suggested also by the Amide I full width at half maximum (28 cm^{-1}), which relates to the strength of the helix, laying here in the medium range. The mode at 1681 cm^{-1} (m) can be assigned to β-turns and to turns of the carbonyl vibration ν(C=O). However, due to the high proportion of arginine in the sequence (28%), it can also be assigned to the side chain vibration of ν_{as} ($CN_3H_5^+$), which absorbs in the 1652-1695 cm^{-1} region. Although the band at about 1638 cm^{-1} (m,s) is usually attributed to a β-sheet structure, it is most likely due to the cumulated contributions of unordered structure, arginine ($CN_3H_5^+$), lysine δ(NH_3^+) and glutamic acid side chains together with β-sheet (as they are a bit high in frequency, they could be indicative of twisted antiparallel structures). For the studied peptides, it is rather unlikely that parallel β-sheet (which absorbs at 1640 cm^{-1}) is present, and this contribution can therefore be discarded.
This fact is confirmed by the Amide II band, which reveals a main mode at about 1544 cm^{-1} (m) generally associated with α-helical structure together with random coil conformations while the presence of β-turns is confirmed by the band at about 1555 cm^{-1} (m). The mode at ca. 1518 cm^{-1} (w) is due to tyrosine residues. The overall broadening of the Amide region might also reflect to the environmental effects. Through-bond coupling and hydrogen bounding may indeed modify the Amide I (and II) frequency of polypeptide to different degrees, as the experiment was run in “close to native” conditions. In sum, MIR investigation of the KARLA peptide reveals the structural organisation of its Amide I band, which secondary structure is essentially composed by α-helical and unfolded molecules.
Although the deconvolution and fit procedures are widely known and are used to assess the secondary structure of proteins (and peptides) in the MIR, additional measurements and techniques (for instance circular dichroism) are sometimes needed to unequivocally resolve the Amide I domain. This can be circumvented in infrared spectroscopy, by switching from MIR to another spectral region as FIR, which can discriminate and identify directly the secondary structure contributions. The concordance of the results in these two spectral domains strengthen and highlight the huge potential of infrared measurements in probing structural parameters and related biological functions.

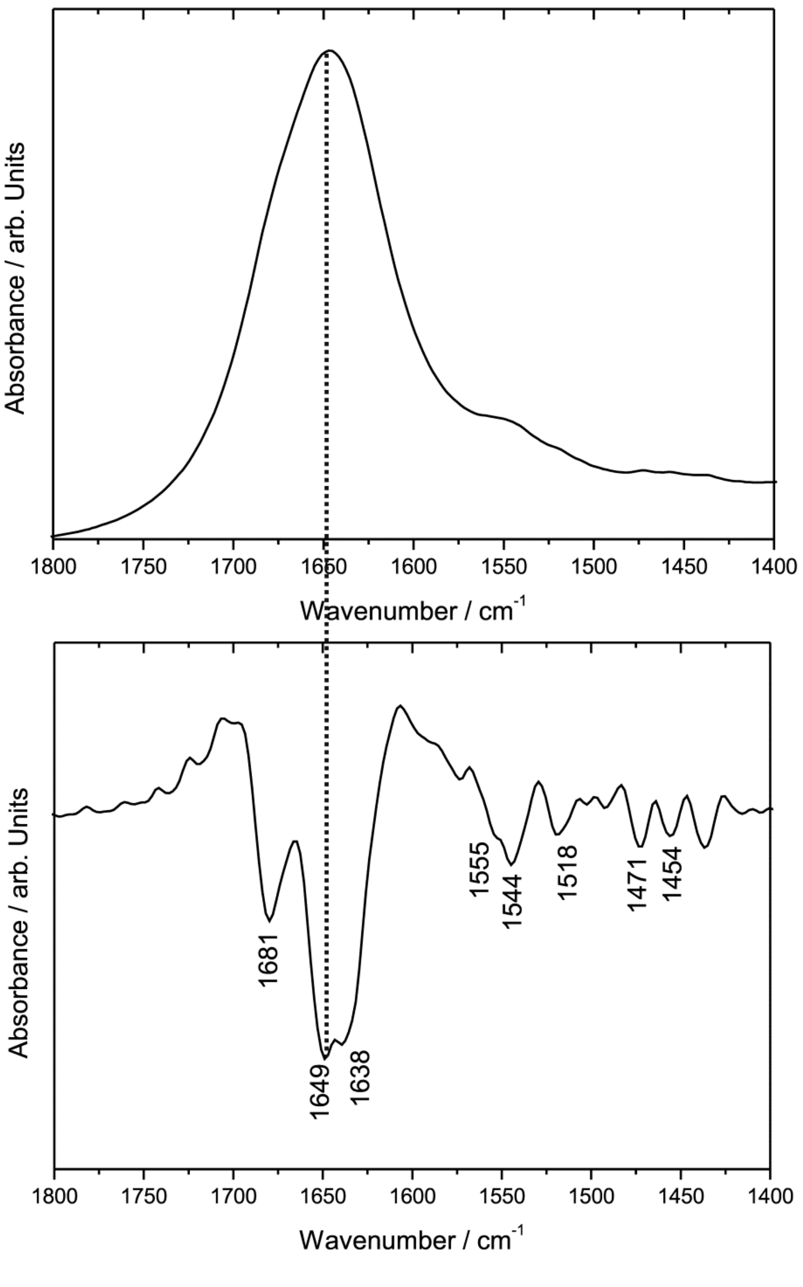

Figure S9. MIR signature of cationic KARLA peptide in PBS buffer solution studied at room temperature (up) and its secondary derivative (down).

3. References

[1] F. Schmid, in *Encycl. Life Sci.*, Macmillan Publishers Ltd, Nature Publishing Group, **2001**, pp. 1–4.

[2] NIST, “http://webbook.nist.gov/cgi/cbook.cgi?ID=C56417&Mask=400, (n.d.).,” **2017**.

[3] G. Upton, I. Cook, in *A Dict. Stat.*, Oxford University Press, **2014**, p. 104-ff.

[4] D. K. Graff, B. Pastrana-Rios, S. Y. Venyaminov, F. G. Prendergast, *J. Am. Chem. Soc* **1997**, *119*, 11282–11294.

[5] H. Fabian, H. H. Mantsch, C. P. Schultz, *Proc. Natl. Acad. Sci. U. S. A.* **1999**, *96*, 13153–13158.

[6] I. Noda, *Biomed. Spectrosc. Imaging* **2015**, *4*, 109–127.

[7] I. Noda, *Appl. Spectrosc.* **1990**, *44*, 550–561.

[8] I. Noda, *J. Am. Chem. Soc.* **1989**, *111*, 8116–8118.

[9] P. A. Madden, R. W. Impey, *Chem. Phys. Lett.* **1986**, *123*, 502–506.

[10] B. M. Fischer, M. Walther, P. Uhd Jepsen, *Phys. Med. Biol.* **2002**, *47*, 3807–3814.

[11] A. Trivella, Y. El Khoury, T. Gaillard, R. H. Stote, N. Merino, F. J. Blanco, P. Hellwig, in *AIP Conf. Proc.*, **2010**, pp. 3–6.

[12] G. Zundel, *J. Mol. Struct.* **1996**, *381*, 23–37.

[13] K. Itoh, T. Shimanouchi, *Biopolymers* **1967**, *5*, 921–30.

[14] K. Itoh, T. Shimanouchi, M. Oya, *Biopolymers* **1969**, *7*, 649–658.

[15] J.-B. Brubach, A. Mermet, A. Filabozzi, A. Gerschel, P. Roy, *J. Chem. Phys.* **2005**, *122*, 184509.

[16] M. Grechko, T. Hasegawa, F. D’Angelo, H. Ito, D. Turchinovich, Y. Nagata, M. Bonn, *Nat. Commun.* **2018**, *9*, DOI 10.1038/s41467-018-03303-y.

[17] K. N. Woods, H. Wiedemann, *J. Chem. Phys.* **2005**, *123*, DOI 10.1063/1.2000238.

[18] R. Hielscher, P. Hellwig, *ChemPhysChem* **2010**, *11*, 435–441.

[19] Y. El Khoury, A. Trivella, J. Gross, P. Hellwig, *ChemPhysChem* **2010**, *11*, 3313–3319.

[20] R. Hielscher, P. Hellwig, *Spectrosc. An Int. J.* **2012**, *27*, 525–532.

[21] G. Zundel, *J. Mol. Struct.* **2000**, *552*, 81–86.

[22] G. D’Angelo, V. Conti Nibali, C. Crupi, S. Rifici, U. Wanderlingh, A. Paciaroni, F. Sacchetti, C. Branca, *J. Phys. Chem. B* **2017**, *121*, 1204–1210.

[23] M. Zhernenkov, D. Bolmatov, D. Soloviov, K. Zhernenkov, B. P. Toperverg, A. Cunsolo, A. Bosak, Y. Q. Cai, *Nat. Commun.* **2016**, *7*, 11575.

[24] J. Bandekar, *Biochim. Biophys. Acta - Protein Struct. Mol. Enzymol.* **1992**, *1120*, 123–143.

[25] A. Barth, *Biochim. Biophys. Acta* **2007**, *1767*, 1073–101.

[26] D. Naumann, *Appl. Spectrosc. Rev.* **2001**, *36*, 239–298.
